# The complex, dynamic SpliceOme of the small GTPase transcripts altered by technique, sex, genetics, tissue specificity, and RNA base editing

**DOI:** 10.3389/fcell.2022.1033695

**Published:** 2022-11-17

**Authors:** Akansha S. Das, Emily C. Sherry, Robert M. Vaughan, Marian L. Henderson, Jacob Zieba, Katie L. Uhl, Olivia Koehn, Caleb P. Bupp, Surender Rajasekaran, Xiaopeng Li, Surya B. Chhetri, Sahar Nissim, Carol L. Williams, Jeremy W. Prokop

**Affiliations:** ^1^ Department of Pediatrics and Human Development, College of Human Medicine, Michigan State University, Grand Rapids, MI, United States; ^2^ Department of Biology, Washington and Jefferson College, Washington, PA, United States; ^3^ Department of Cell and Molecular Biology, Grand Valley State University, Allendale, MI, United States; ^4^ The Department of Biology, Calvin University, Grand Rapids, MI, United States; ^5^ Genetics and Genome Sciences Program, BioMolecular Science, Michigan State University, East Lansing, MI, United States; ^6^ Department of Pharmacology and Toxicology, Medical College of Wisconsin, Milwaukee, WI, United States; ^7^ Medical Genetics, Spectrum Health and Helen DeVos Children’s Hospital, Grand Rapids, MI, United States; ^8^ Department of Pediatric Critical Care Medicine, Helen DeVos Children’s Hospital Spectrum Health, Grand Rapids, MI, United States; ^9^ Office of Research, Spectrum Health, Grand Rapids, MI, United States; ^10^ Department of Biomedical Engineering, Johns Hopkins University, Baltimore, MA, United States; ^11^ Genetics and Gastroenterology Divisions, Brigham and Women’s Hospital, Harvard Medical School, Boston, MA, United States; ^12^ Dana-Farber Cancer Institute, Boston, MA, United States; ^13^ Harvard-MIT Division of Health Sciences and Technology, Cambridge, MA, United States; ^14^ Department of Pharmacology and Toxicology, Michigan State University, East Lansing, MI, United States

**Keywords:** small GTPase, splicing aberrations, expression, isoforms, protein modeling, RNA modifications, cancer

## Abstract

The small GTPase family is well-studied in cancer and cellular physiology. With 162 annotated human genes, the family has a broad expression throughout cells of the body. Members of the family have multiple exons that require splicing. Yet, the role of splicing within the family has been underexplored. We have studied the splicing dynamics of small GTPases throughout 41,671 samples by integrating Nanopore and Illumina sequencing techniques. Within this work, we have made several discoveries. 1). Using the GTEx long read data of 92 samples, each small GTPase gene averages two transcripts, with 83 genes (51%) expressing two or more isoforms. 2). Cross-tissue analysis of GTEx from 17,382 samples shows 41 genes (25%) expressing two or more protein-coding isoforms. These include protein-changing transcripts in genes such as *RHOA*, *RAB37*, *RAB40C*, *RAB4B*, *RAB5C*, *RHOC*, *RAB1A*, *RAN*, *RHEB*, *RAC1*, and *KRAS*. 3). The isolation and library technique of the RNAseq influences the abundance of non-sense-mediated decay and retained intron transcripts of small GTPases, which are observed more often in genes than appreciated. 4). Analysis of 16,243 samples of “Blood PAXgene” identified seven genes (3.7%; *RHOA*, *RAB40C*, *RAB4B*, *RAB37*, *RAB5B*, *RAB5C*, *RHOC*) with two or more transcripts expressed as the major isoform (75% of the total gene), suggesting a role of genetics in altering splicing. 5). Rare (*ARL6*, *RAB23*, *ARL13B*, *HRAS*, *NRAS*) and common variants (*GEM*, *RHOC*, *MRAS*, *RAB5B*, *RERG*, *ARL16*) can influence splicing and have an impact on phenotypes and diseases. 6). Multiple genes (*RAB9A*, *RAP2C*, *ARL4A*, *RAB3A*, *RAB26*, *RAB3C*, *RASL10A*, *RAB40B*, and *HRAS*) have sex differences in transcript expression. 7). Several exons are included or excluded for small GTPase genes (*RASEF*, *KRAS*, *RAC1*, *RHEB*, *ARL4A*, *RHOA*, *RAB30*, *RHOBTB1*, *ARL16*, *RAP1A*) in one or more forms of cancer. 8). Ten transcripts are altered in hypoxia (*SAR1B*, *IFT27*, *ARL14*, *RAB11A*, *RAB10*, *RAB38*, *RAN*, *RIT1*, *RAB9A*) with *RHOA* identified to have a transient 3′UTR RNA base editing at a conserved site found in all of its transcripts. Overall, we show a remarkable and dynamic role of splicing within the small GTPase family that requires future explorations.

## Introduction

Small GTPases are indispensable components of cell communication, intracellular trafficking, vesicular trafficking, and cell migration (Bourne et al., 1991; Mizuno-Yamasaki et al., 2012; Lawson and Ridley, 2018). The family is deeply conserved throughout eukaryotes (Jékely, 2003; Boureux et al., 2007), with multiple functional subfamily members in plants (Vernoud et al., 2003), Bivalvia genomes (Li et al., 2015), and trypanosomes (Field, 2005). The GTPase factors found in bacteria have been suggested to play a role in RNA functional regulation (Caldon et al., 2001), highlighting how GDP/GTP biology impacts all life forms through RNA.

The small GTPases comprise the RAS superfamily, containing RAS, RHO, RAB, RAP, RIT, and ARF members (Reuther and Der, 2000; Colicelli, 2004). These members contain GDP and GTP-bound forms that can be regulated by guanine nucleotide exchange factors (GEFs) and GTPase activating proteins (GAPs) (Bar-Sagi and Hall, 2000). Two areas of RAS member genetics have been a focus over the past decade, their role in rare diseases and their implications in cancer. RASopathies are derived from germline changes in RAS and MAPK genes within 1:1,000 births, displaying vascular, cardiac, bone, and cell proliferation phenotypes (Rauen, 2013; Aoki et al., 2016; Tidyman and Rauen, 2016). Many RASopathies also have complex developmental delay and autistic spectrum disorder (ASD), highlighting the role of small GTPase genes in brain development (Ba et al., 2013; Adviento et al., 2014). The gene family has been extensively studied for its role in cancer development, progression, and metastasis (Vega and Ridley, 2008; Kazanietz and Caloca, 2017), with recent advances moving toward targeted treatments based on the detection of small GTPase variants (Prieto-Dominguez et al., 2019).

Small GTPases contain a diverse set of exons with propensity for alternative splicing that can impact biology, including neurodevelopmental disorders and cancer (Lee et al., 2022). Within cancer, splicing changes in small GTPases have been noted for genes including *NRAS*, *KRAS*, *HRAS*, and *RAC1* (Rásó, 2020). The Neuroblastoma RAS (*NRAS*) gene has five expressed isoforms, with two of the isoforms elevated in melanoma patients (Duggan et al., 2019; Yan et al., 2019). The Kirsten rat sarcoma virus gene (*KRAS*) has four splice variants, with KRAS-201 (KRAS4a) and KRAS-202 (KRAS4b) having significantly different roles in the development of cancer due to their changes in the C-terminal region (Plowman et al., 2006; Abubaker et al., 2009; Chakrabarti et al., 2016). The Harvey Rat sarcoma virus gene (*HRAS*) has a retained intron transcript and an alternative C-terminal splice site that influence total protein levels and interaction partners (Cohen et al., 1989; Guil et al., 2003). Ras-related C3 botulinum toxin substrate one gene (*RAC1*) has two isoforms that change the sequence of the protein (RAC1 and RAC1B), with differential risks to cancer progression (Jordan et al., 1999; Melzer et al., 2019).

**FIGURE 1:**
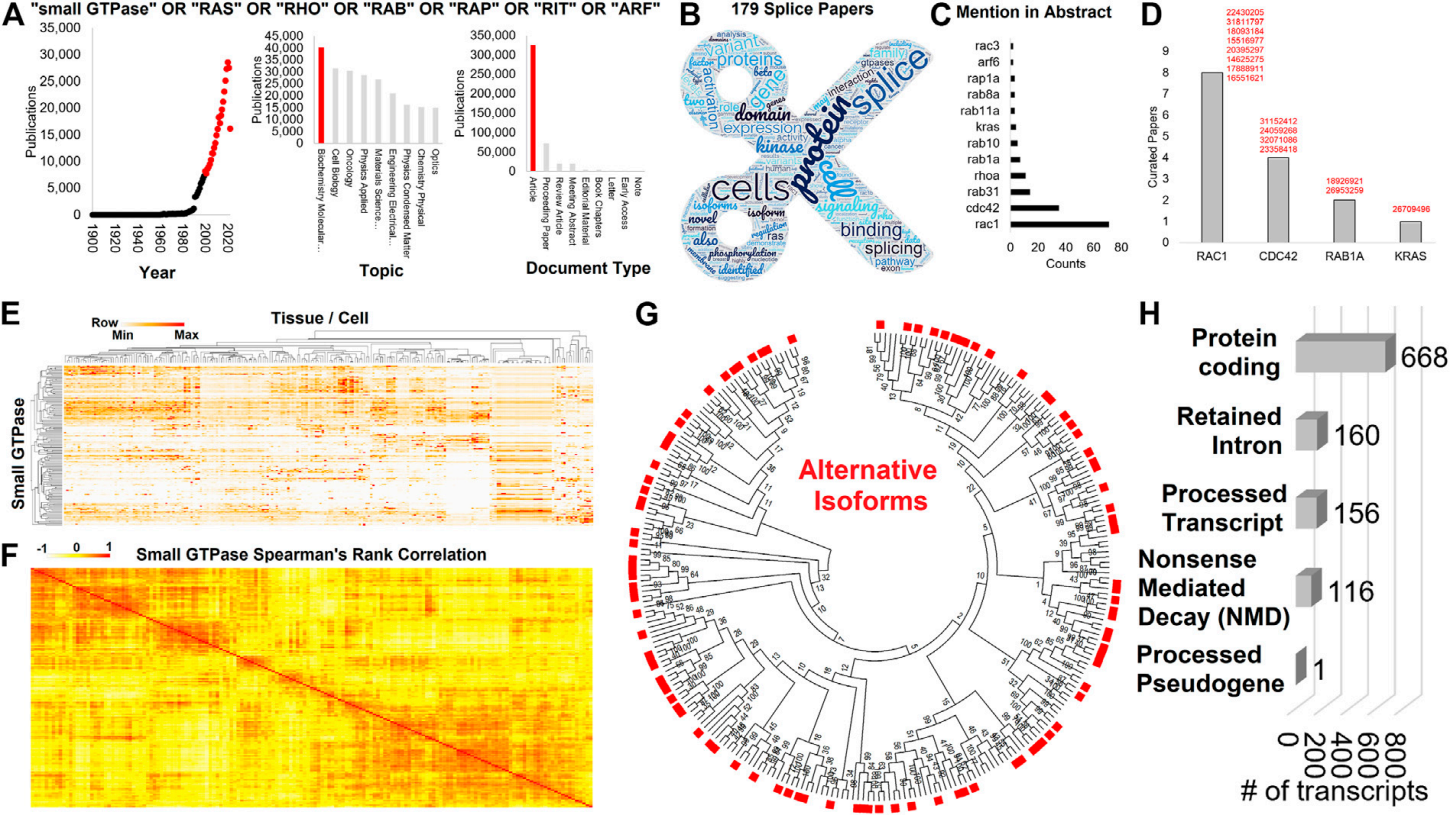
Expression and isoforms of small GTPase family genes. **(A)** Web of Science extraction for small GTPase OR RAS OR RHO OR RAB OR RAP OR RIT OR ARF followed by analysis of papers for year, topic, and document type. Those categories in red were selected for further analysis. **(B)** Word cloud of top terms extracted from the abstract of the 179 papers using panel A inclusion and the word splice. **(C)** The number of times any small GTPase gene is included in the abstracts of **(B)**. **(D)** The number of curated manuscripts for small GTPase genes that include isoform information. The PMID codes are listed next to each gene in red. **(E)** Heat map of the Human Protein Atlas expression for each small GTPase family member (*y*-axis) across multiple human tissue and cell (*x*-axis). Clustering is based on one minus Spearman’s rank correlation. Coloring is based on the z-score for each gene, with the middle level in yellow and the highest in red. **(F)** Spearman’s rank correlation for each of the small GTPase family members to each other based on **(A)** expression. **(G)** A maximum likelihood tree for all UniProt annotated isoforms of small GTPase members. Red boxes are alternative protein isoforms from the primary annotated form (no red box). **(H)** Annotated Biotypes for small GTPase isoforms from Gencode 39.

While the majority of literature has focused on oncogenic or RASopathy splice variants of small GTPases, it is also important to understand small GTPase splicing in various tissues and how changes differ by age, sex, RNA sequencing strategy (ex: poly A vs. total RNA sequencing), or environmental factors. Of the 432,477 small GTPase annotated papers on Web of Science (small GTPase OR RAS OR RHO OR RAB OR RAP OR RIT OR ARF), a total of 179 papers include the word “splice”, are from 2000 to 2022, annotated as biochemistry and molecular biology, and are original articles (Figures 1A,B, https://doi.org/10.6084/m9.figshare.21381900.v1 tab Figure 1B). Abstracts from these papers were extracted for gene symbols (Figure 1C) and manually curated for splicing of small GTPase genes (Figure 1D). Outside of *RAC1* and *KRAS* already mentioned, there are multiple papers on *CDC42* (Nishimura and Linder, 2013, 2019; Wirth et al., 2013; Endo et al., 2020) and *RAB1A* (Deng et al., 2009; Schöppner et al., 2016) isoforms. Some evidence also exists for alternative splicing of RHOA in gastric carcinoma (Miyamoto et al., 2018). In this context, understanding the distribution of small GTPase isoforms, outside of those previously annotated within the literature, under normal physiologic conditions can improve understanding of this splicing impact on other pathologies.

The human genome encodes >250,000 transcripts represented by diverse biotypes (Prokop et al., 2022). Of these transcripts, 37% are polyadenylated and code for proteins (protein coding), which account for most studied/published transcripts. There are also 13% of transcripts where an intron is retained that disrupts the open reading frame (retained intron) and 8% that have a spliced form that results in non-sense-mediated decay (NMD). NMD is due to the inclusion of a premature stop codon followed by several spliced exons after the stop, where these products are widely believed to be degraded to prevent the production of abnormal protein products (Maquat, 2004). Alternative splicing allows for the potential of many different transcripts to be made from the same gene or for a transcript to result in no protein produced (Wang et al., 2015; Mapleson et al., 2018). Splicing increases the complexity of the proteome from ∼20 thousand protein-coding genes to nearly 100 thousand different proteins, with cellular-specific splicing dynamics (Modrek and Lee, 2002; Stamm et al., 2005). While many of the cellular dynamics of alternative splicing are not fully understood, the regulation of splicing machinery through gene expression, posttranslational modification, and RNA-protein interactions can modify the inclusion or exclusion of an exon within a gene to drive cellular-specific splicing outcomes (Matlin et al., 2005). The dysregulation of these splicing pathways through environmental factors impacting the splicing machinery genes/proteins or genomic variants altering the ability of splice machinery to interact with RNA at splice junctions can result in cellular dysfunction that can yield diseases (Tazi et al., 2009). With the advancement of sequencing and proteomic techniques and the investment in larger-scale data collection, much of the splicing dynamics for various genes can now be explored.

Within this work, we describe an analysis of splicing for the small GTPase family using 41,671 samples. These include 92 samples of long read technologies capable of complex exon maps, 17,382 samples of GTEx with biological variables (tissue, sex, and age), 16,243 samples of multiple methods for blood biomarker analysis, 7,934 case and control matched samples of the Cancer Genome Atlas (TCGA) for 16 cancer types, and 20 samples from a hypoxia exposed cell culture experiment using three independent RNAseq technologies. Throughout these studies, we focus on transcripts that alter protein sequence, annotating transcript biotypes that do not code for proteins, and genetic variants’ role in splicing outcomes. Thus, we provide a robust analysis of the small GTPase SpliceOme, highlighting the need for future investments in splicing biology insights.

## Results and discussions

### Small GTPase genes and resulting isoforms

There are 162 annotated small GTPase genes within UniProt (https://doi.org/10.6084/m9.figshare.20371842, UniProt tab), with 159 also identified within the Human Protein Atlas (HPA, https://doi.org/10.6084/m9.figshare.20371842, HPA tab). These genes have a broad expression profile over human tissues and cells (Figure 1E), showing clustering around functional groups of cell types. Additionally, these small GTPase genes have expression correlations with each other (Figure 1F), suggesting that groups of small GTPases work cohesively in cell and tissue signaling. An analysis of all protein-coding sequences within UniProt for these 162 small GTPase genes shows that throughout the entire family, alternatively spliced isoforms exist (Figure 1G). Not only can splicing alter the form of these proteins, but it can result in additional transcripts that have retained introns, processed transcripts, transcripts that may undergo NMD or processed pseudogenes that likely do not result in proteins (Figure 1H, https://doi.org/10.6084/m9.figshare.21381900.v1 tab Figure 1H). Yet, as of 2022, we are unaware of any works that systematically characterize the splicing dynamics of the small GTPase family.

### GTEx multiple tissue small GTPase splicing

**FIGURE 2:**
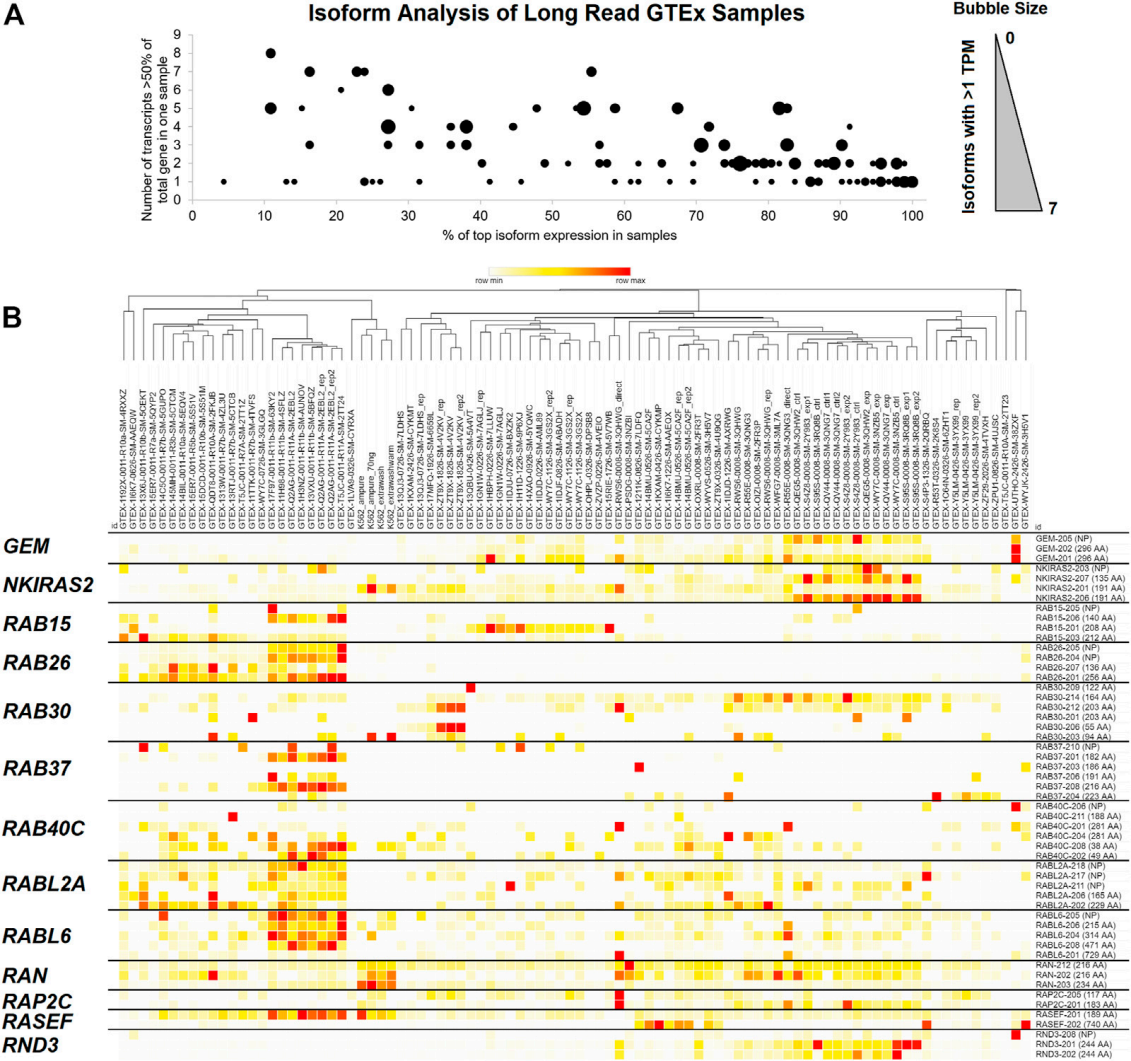
GTEx long-read sequencing data for small GTPase isoforms. **(A)** Analysis of the small GTPase genes for protein-coding isoform expression in Nanopore-based long-read sequencing, where each spot corresponds to one small GTPase gene. The *x*-axis shows each gene’s average % expression of the top expressed isoform. The *y*-axis shows the number of transcripts greater than 50% of a gene expression in any sample. The dot size corresponds to the number of protein-coding isoforms within a gene with an average greater than one transcript per million sequences (TPM). Values with zero are not shown, and the maximum number of isoforms is seven within one gene. **(B)** Heat map of Nanopore-based long read samples of GTEx for several genes that have expressed multiple isoforms. The top shows each Nanopore sample listed as a column with a dendrogram generated as one minus Spearman’s rank correlation. The right shows isoforms for each row. Coloring is based on the z-score for each gene across all samples, with the middle level in yellow and the highest in red.

We began our analysis by taking one of the largest databases of long-read sequencing of transcripts, the GTEx Nanopore expression analysis (quantification_flair_filter.tpm.txt.gz), and extracting the isoform level expression map of the small GTPase genes for 92 samples (https://doi.org/10.6084/m9.figshare.20371842, GTEx Long Read tab). Long-read Nanopore-based data represents full-length sequencing transcripts, yielding higher accuracy of splicing insights than short-read technologies. There were 162 small GTPase genes with 874 total transcripts annotated. A total of 154 small GTPase genes were annotated with at least one protein-coding transcript above one transcript per million (TPM) within a sample, with an average of two transcripts per gene (Figure 2A, size of bubble, https://doi.org/10.6084/m9.figshare.21381900.v1 tab Figure 2A). One gene, *RHOC*, had seven annotated protein-coding transcripts expressed higher than one TPM (Figure 2A, size of bubble). Three genes had six isoforms (*MRAS*, *RAB34*, *RABL2B*), five genes with five (*ARFRP1*, *ARL4A*, *NKIRAS1*, *NKIRAS2*, *SAR1A*), fourteen genes with four isoforms, twenty-two with three isoforms, thirty-eight with two isoforms, and seventy-one with one protein-coding isoform greater than one TPM. Of those genes with protein-coding transcripts, there is a balance of how highly expressed the top isoform is (Figure 2A, *x*-axis) relative to how many transcripts any sample has expressed (Figure 2A, *y*-axis).

Curation of several genes with isoforms that change protein sizes can be seen in Figure 2B. *GEM* has two isoforms that result in a 296 amino acid (AA) protein and one that does not code for a protein. *NKIRAS2* has two isoforms for a 191 AA protein, one for a 135 AA, and one that does not code for a protein. *RAB15* has 140, 208, and 212 AA coding transcripts; *RAB26* has 136 and 256 AA coding transcripts; *RAB30* has 55, 94, 122, 164, and 203 AA coding transcripts; *RAB37* has 182, 186, 191, 216, and 223 AA coding transcripts; *RAB40C* 38, 49, 188, 281 AA coding transcripts; *RABL2A* has 165 and 229 AA coding transcripts; *RABL6* has 215, 314, 471, and 729 AA coding transcripts; *RAN* has 216 and 234 AA coding transcripts; *RAP2C* 117 and 183. This long-read analysis strongly supports that alternative splicing can result in different protein sequences for many small GTPase genes.

**FIGURE 3:**
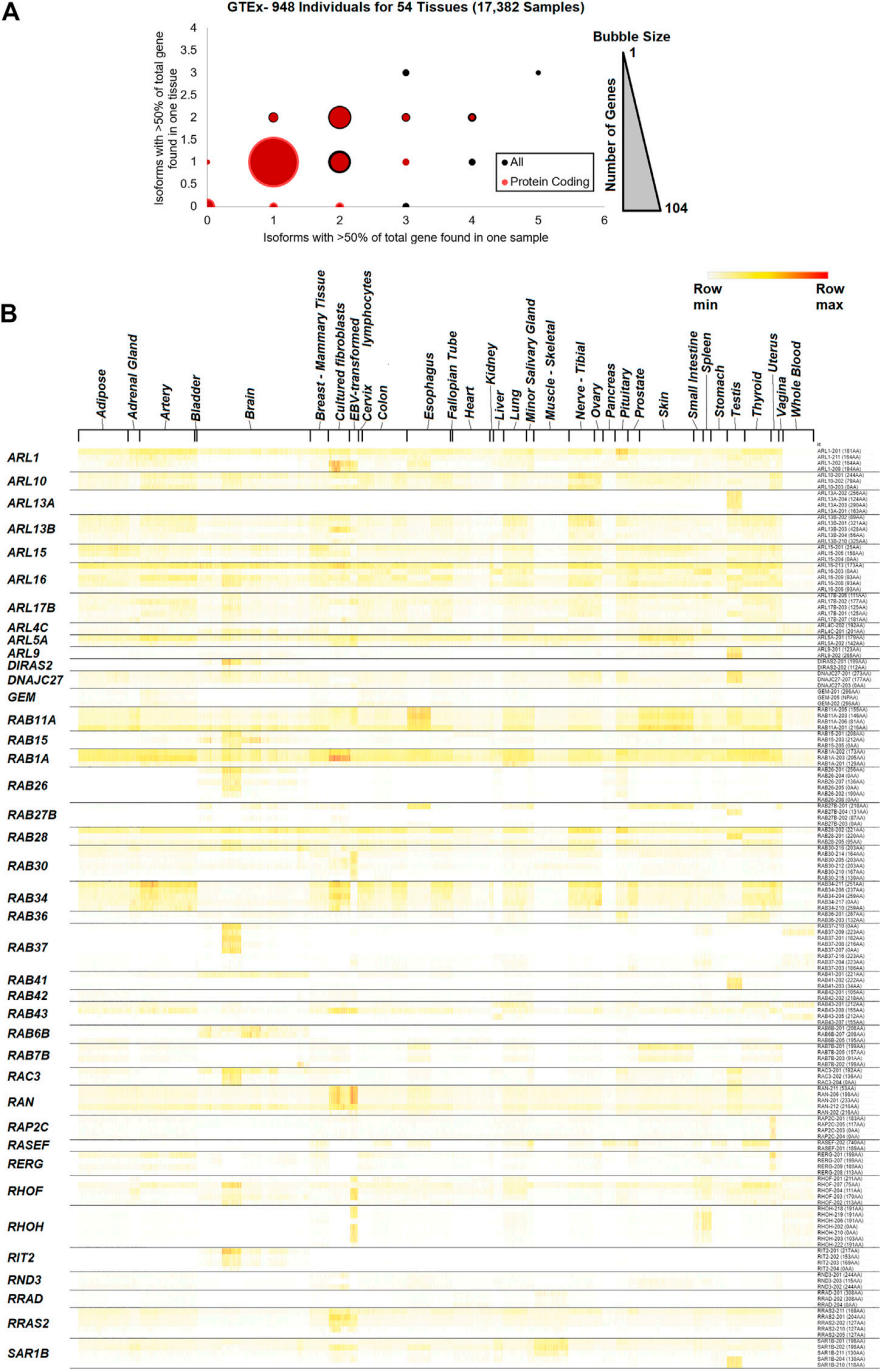
GTEx tissue-specific data for small GTPase isoforms. **(A)** The number of isoforms across samples and tissues. The *x*-axis shows the number of isoforms that account for at least 50% of the gene expression in one sample, while the *y*-axis shows the number of isoforms that account for at least 50% of the gene expression in one tissue. The bubble size represents the number of isoforms at each x and y level, with a maximum of 104. The red spots represent protein-coding transcripts, and the black spots all transcripts. **(B)** Heat map of transcript expression for several small GTPase genes with more than one isoform expressed across tissues. Sample tissues are labeled on the top and isoforms for each gene on the right. Coloring is based on the z-score for each gene, with the middle level in yellow and the highest in red.

Therefore, we expanded into a more extensive short-read, Illumina-generated annotation of small GTPase splicing from GTEx (GTEx_Analysis_2017-06-05_v8_RSEMv1.3.0_transcript_tpm.gct.gz). This dataset contained 17,382 samples from 948 individuals for 54 different tissues. A total of 996 transcripts were identified for 159 small GTPase genes. Analysis of common isoforms (>50% of all transcripts encoded by the gene) within a sample relative to all samples for a tissue shows that most of the small GTPase genes (88 genes based on all transcripts and 104 genes based only on protein-coding) have only one isoform that accounts for the majority of expression (Figure 3A, https://doi.org/10.6084/m9.figshare.21381900.v1 tab Figure 3A). Forty-one genes with at least two protein-coding isoforms are found in a sample or tissue >50% of transcripts for the encoded gene (https://doi.org/10.6084/m9.figshare.20371842, GTEx isoforms tab), with 17 found with values of two on each axis. *RAB43* has four different protein-coding isoforms with >50% within one sample, while *RERG*, *RAB41*, *RIT2*, and *RAB26* have three protein-coding isoforms. A representative heat map of 40 small GTPase genes with greater than two isoforms is shown for the diverse tissues of the GTEx database (Figure 3B, https://doi.org/10.6084/m9.figshare.21381900.v1 tab Figures 3A,B Tissue).

### Blood-based dynamics of small GTPase splicing

**FIGURE 4:**
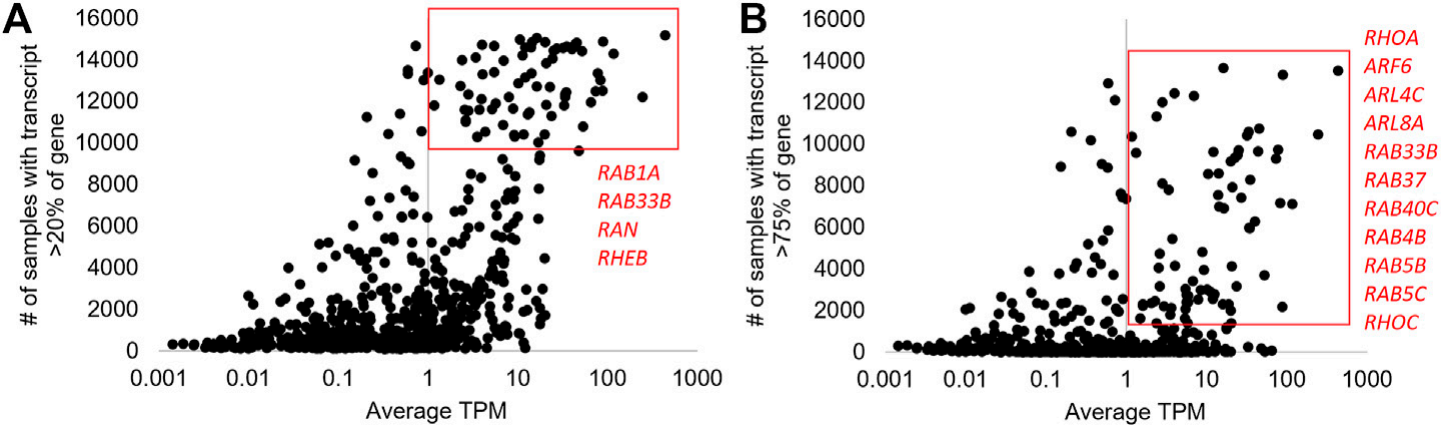
“Blood PAXgene” based changes in small GTPase isoforms. **(A)** The Analysis of 16,243 samples from 116 NCBI BioProjects for isoforms of small GTPase genes. The *x*-axis shows the average transcripts per million (TPM) for each isoform, and the *y*-axis shows the number of samples where that isoform is greater than 20% of the total gene expression. The red box is around those isoforms with >10,000 samples and one TPM; the four genes with more than one isoform are labeled in red. **(B)** The same plot in **(A)** shows the *y*-axis for samples greater than 75%. The red box is around those with >2,000 samples and 1 TPM, with the 11 genes having >1 isoform in the box labeled in red.

As blood is an easily collectible material through the use of PAXgene tubes, and one in which our group has built extensive bioinformatics analyses (Prokop et al., 2020; Prokop et al., 2021; Bauss et al., 2021; Gupta et al., 2021), we curated small GTPase expression within 16,243 samples from 116 BioProjects for “Blood PAXgene.” Samples from this database represent healthy individuals and patients with various pathologies, capturing all known human samples from a single collection tube type (https://doi.org/10.6084/m9.figshare.21381900.v1 tabs SRA PAXgene Blood BioProjects, SRA PAXgene Blood Samples, Figure 4). This represents an array of isolation and sequencing strategies, including polyA RNAseq, total (ribosomal reduction ± globin reduction), or small RNA isolations such as miRNA. A total of 1,209 isoforms from 162 genes were obtained for small GTPases (https://doi.org/10.6084/m9.figshare.20371842, Blood isoforms tab).

There were 70 transcripts of small GTPases with an average of one TPM (>10,000 samples with >20% of the genes’ total expression level, Figure 4A), representing common spliced versions of proteins throughout blood samples. Two genes have multiple transcripts that result in the same protein sequence (*RAB6A-201/202*- 208AA; *RAB9A-202/203*- 201AA), and one gene has a protein-coding and non-coding transcript (*RAB4A-201*- 218AA, *RAB4A-202*- no protein). Four genes have two different protein-coding isoforms including *RAB1A* (*RAB1A-203*- 205AA, *RAB1A-202*- 173AA), *RAB33B* (*RAB33B-203*- 277AA, *RAB33B-201*- 229AA), *RAN* (*RAN-206*- 198AA, *RAN-211*- 53AA), and *RHEB* (*RHEB-201*- 184AA, *RHEB-204*- 79AA). These suggested genes have multiple protein forms found expressed in a large portion of blood samples.

A more rigorous inclusion criterion of transcripts that account for a higher % of total transcripts (75%) but with fewer samples (>1,000) identifies a different set of genes with multiple protein-coding transcripts (Figure 4B). Thus, panel A represents transcripts seen in many individuals at high levels, while panel B represents transcripts observed in some individuals. Of the 80 transcripts that reach inclusion criteria, *RHOA* is the only gene with three transcripts (*RHOA-202/209*- 193AA, *RHOA-207*- 109AA). Two genes have a protein-coding and non-coding transcript identified including *RAB40C* (*RAB40C-206*- no protein, *RAB40C-204*- 281AA) and *RAB4B* (*RAB4B-204*- no protein, *RAB4B-201*- 213AA). Four genes have multiple isoforms of the same protein, including *RAB37* (223AA), *RAB5B* (215 AA), *RAB5C* (216 AA), and *RHOC* (193 AA). Four genes have multiple protein sizes, including *ARF6* (46/175 AA), *ARL4C* (192/201 AA), *ARL8A* (147/186 AA), and *RAB33B* (229/277 AA). This analysis suggests that genetic variants within samples may influence the splicing outcomes of several small GTPase genes, which will be addressed later. In the future, we hope some of these isoforms may have discovery potential as pathology biomarkers. However, this will require the growth of transcriptomic databases such as this with more clinical annotations. Overall, the GTEx and blood PAXgene datasets suggest that small GTPase genes can have extensive alternative splicing. Therefore, we set out to better understand how various factors can modulate splicing within the gene family.

### Protein altering isoform of small GTPases

**FIGURE 5:**
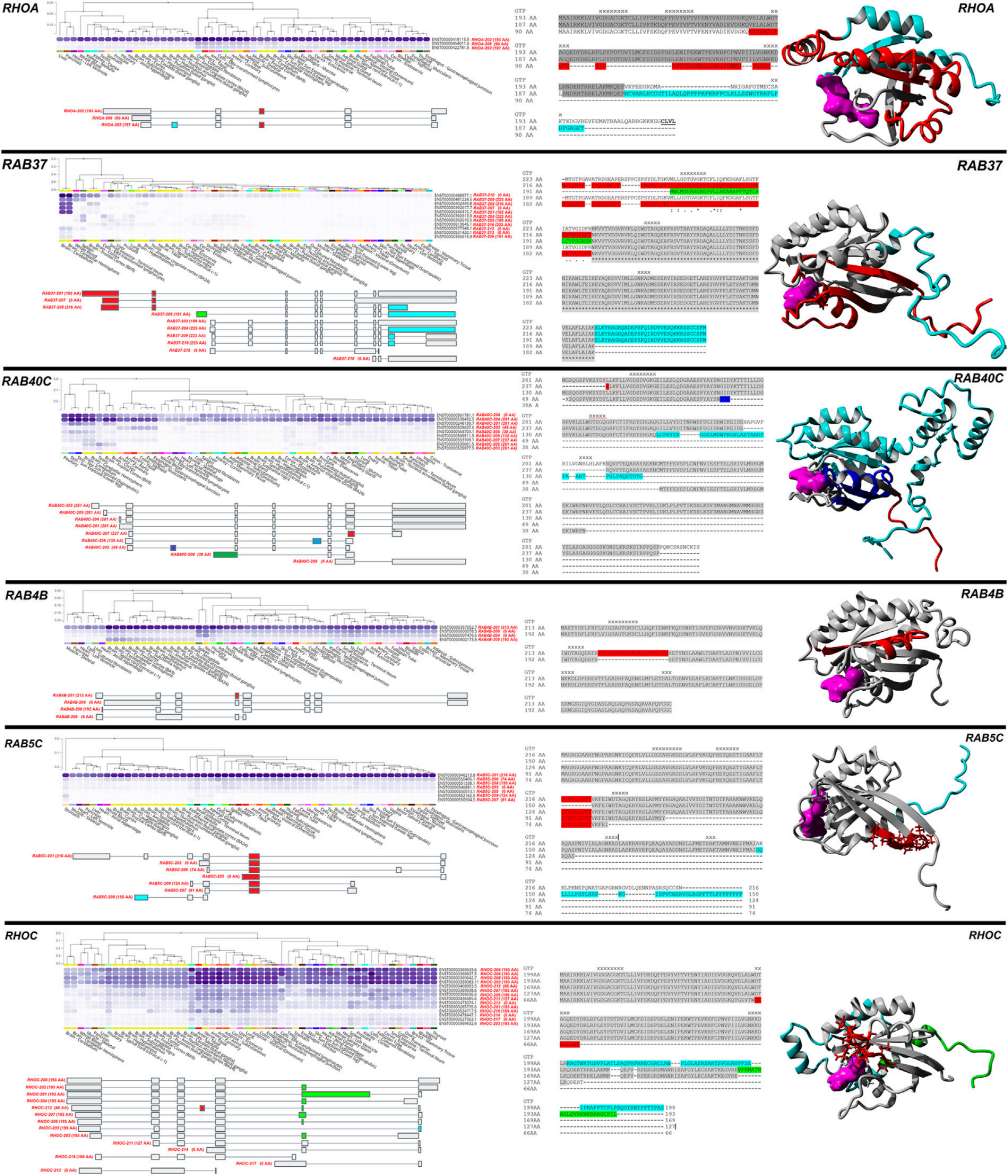
Examples of protein-changing isoforms with tissue-based dynamics. Six genes/proteins are shown (*RHOA*, *RAB37*, *RAB40C*, *RAB4B*, *RAB5C*, *RHOC*), with a black line separating them. For each gene/protein, the top left panel shows different isoform expressions in human tissues, with dark blue the highest expression. Ensemble identifiers are labeled in black for each isoform and gene transcript with AA size in brackets labeled in red. This image is modified from the GTEx website for each gene. Below the GTEx expression data is isoform splicing, with colored regions changing the protein sequence. In the middle of each gene are the sequence alignments for the different proteins with color highlights corresponding to the splicing map. The GTP/GDP binding sites are marked with an X. To the right is a protein model, with colors identifying the sites in the alignment. GTP/GDP are colored magenta.

Six genes (*RHOA*, *RAB37*, *RAB40C*, *RAB4B*, *RAB5C*, *RHOC*) with different protein-coding isoforms having diverse tissue expression based on GTEx were selected as examples (Figure 5). *RHOA* has three transcripts coding for three proteins. The *RHOA-202* transcript codes for a 193 AA protein that is ubiquitously expressed, while transcript RHOA-206 (90 AA) and RHOA-203 (187 AA) are highest expressed in fibroblasts and arteries. The 187 AA and 193 AA transcripts have an additional intron from the 90 AA transcript that changes the frame of the protein after the shared N-terminal region (red), and the 187 AA transcript has one additional exon than the others that changes the c-terminal sequence of the protein (cyan). *RAB37* has an array of different transcripts that do or do not code for protein sequences. Four transcripts (*RAB37-207-210*) are highly expressed in the brain cerebellum and cerebellar hemisphere, while three (*RAB37-201/202/204*) are not expressed in those tissues but are found broadly in other tissues. Of the five isoforms expressed that code for different proteins, two splicing differences change the N-terminal segment of RAB37 (red and green), and one splicing difference changes the C-terminal region (cyan).

In the case of *RAB40C,* both *RAB40C-204* coding for a 281 AA protein and *RAB40C-206* not coding for a protein are ubiquitously expressed, while an array of altered protein transcripts, including small proteins that are likely NMD regulated, have diverse expression. One splice difference alters the C-terminal segment (red), while other splicing differences cause frameshift changes that remove large portions of RAB40C (blue and cyan). For *RAB4B,* the *RAB4B-201* isoform resulting in a 213 AA protein is ubiquitously expressed, while the exclusion of an exon (red) decreased the protein size to 192 AA by removing an alpha helix segment in the middle of RAB4B and is expressed in various brain regions. Two additional transcripts of *RAB4B* (*RAB4B-204/206*) can be found in tissues such as spleen and tibial nerve but do not code for a protein. For *RAB5C,* isoform 201 is ubiquitously expressed, with a one exon exclusion (red) altering a middle segment. Another splicing difference causes a frameshift variant (cyan), where each isoform is expressed in only a few tissue types. Finally, in *RHOC*, the combination of four isoforms (202/204/206/208), all coding for a 193 AA protein are ubiquitously expressed, while several splicing differences (red, cyan, green) result in frameshift changes expressed in only a few tissue types.

**FIGURE 6:**
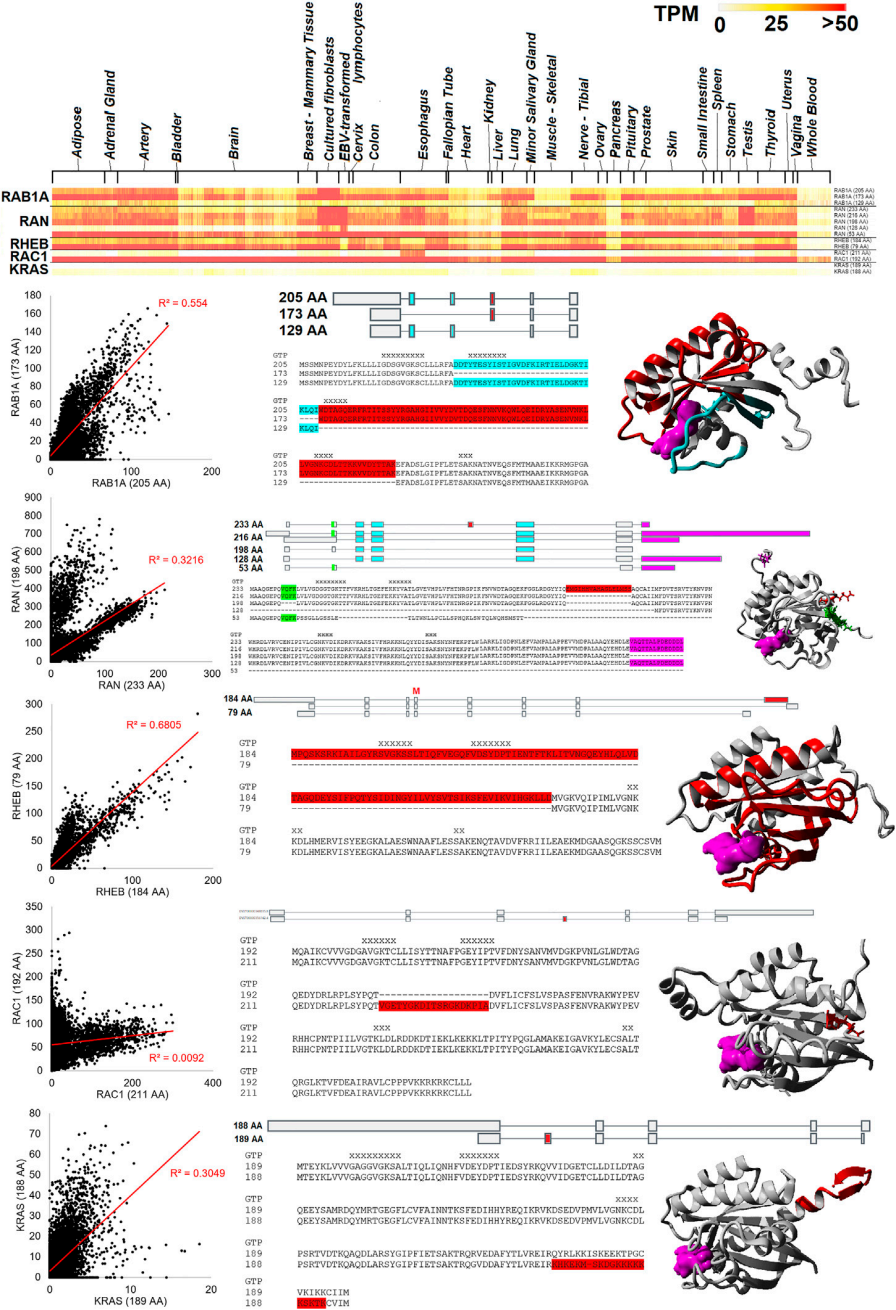
Small GTPase splicing differences seen in many tissues. The top panel shows a heat map of *RAB1A*, *RAN*, *RHEB*, *RAC1*, and *KRAS* isoforms for GTEx tissues. Color is based on transcripts per million (TPM) values, where yellow is 25 TPM and red is >50 TPM. Below the heat map are details for each of the five genes. The left panel shows the values of the top protein isoforms in the 16,243 “Blood PAXgene” samples, where the *x* and *y* axes show different isoform values. The red line represents the best fit line with an R-squared correlation shown in red. The middle shows the isoform map and sequence alignment with variable regions identified with colors. On the far right is the protein model, with colors similar to the alignment and GTP/GDP in magenta.

Five small GTPase genes have two or more ubiquitously expressed protein-coding isoforms, including *RAB1A*, *RAN*, *RHEB*, *RAC1*, and *KRAS* (Figure 6). As shown on the heat map for Figure 6, these different protein isoforms are expressed throughout the tissues of GTEx. Moreover, each gene shows expression of both isoforms within our blood RNAseq datasets. However, some isoforms have a high correlation with expression levels in the blood, such as *RHEB* (R-squared of 0.68) and *RAB1A* (0.55). Others show a slight correlation, such as *RAN* (0.32), where it has a bimodal distribution. Two genes show little correlation, *KRAS* (0.30) and *RAC1* (0.01). *RAB1A* has three highly expressed protein forms (205, 173, and 129 AA), where three exons alter the middle of the protein sequence, including two different GTP coordination sites. *RAN* has five different protein-coding forms (233, 216, 198, 128, and 53 AA), with differences found outside the GTP binding sites and most of the secondary structure. *RHEB* has two primary protein-coding forms (184 and 79 AA) that remove two GTP binding sites of the C-terminus. *RAC1* has two primary protein-coding forms (211 and 192 AA) that result in the insertion of a loop segment. *KRAS* has two of the most well-studied protein forms (189 and 188 AA) that result from an included exon that causes a frameshift. Both isoforms have a similar CAAX motif in the last four AAs, highlighting how alternative frames have been selected for similar functional motifs.

### Biotype annotation of small GTPase splicing

**FIGURE 7:**
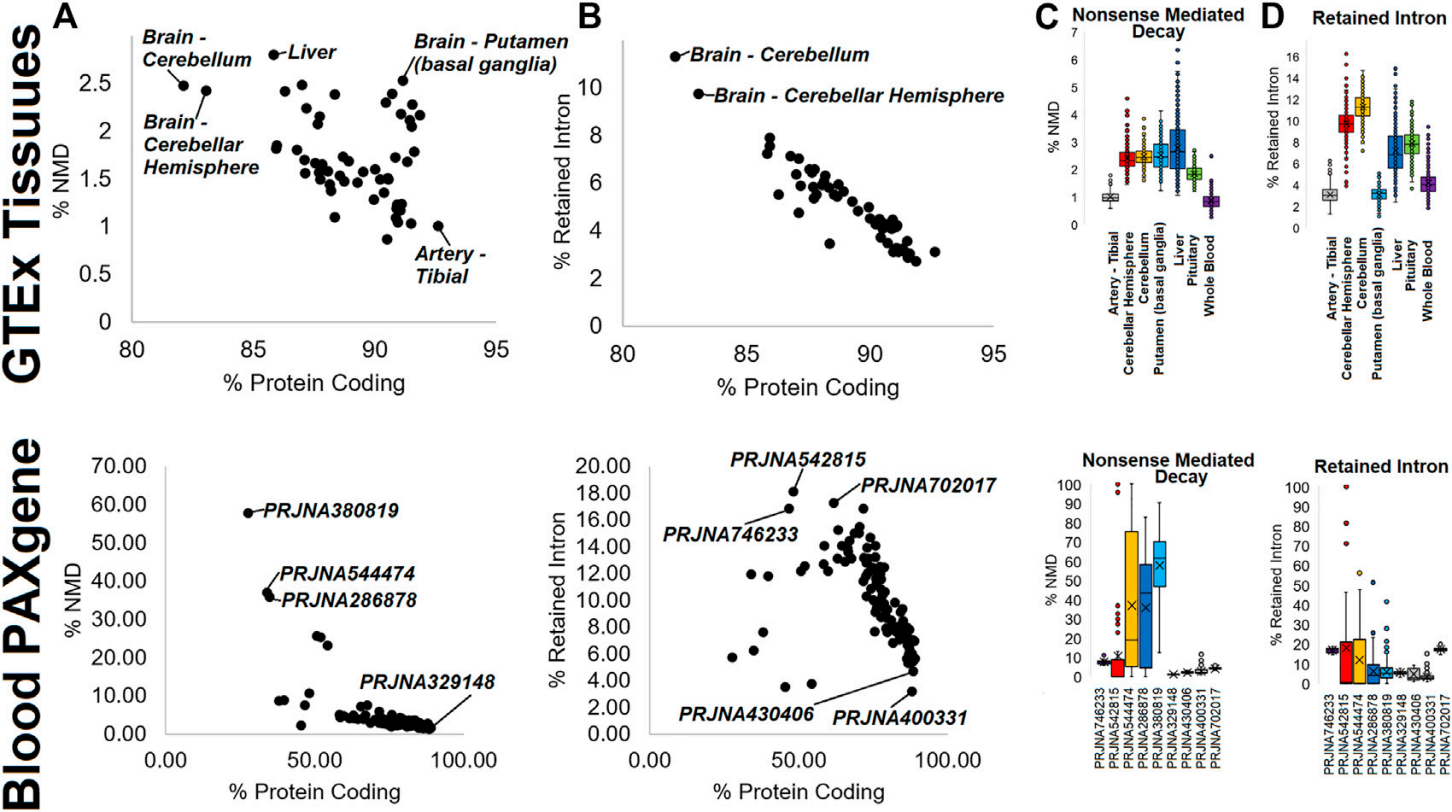
Biotype annotations in mapping data. The top four panels show data for GTEx, while the bottom four panels show data for “Blood PAXgene.” **(A,B)** Analysis for each tissue of GTEx (top) or BioProject of the SRA (bottom) for the percent of a small GTPase genes expression for protein-coding (*x*-axis) vs. either nonsense-mediated decay (NMD, **(A)** or retained intron isoforms **(B)**. **(C,D)** Box and whisker plot for several outlier tissues (top) or BioProjects (bottom) of % of isoforms for each gene mapping to nonsense-mediated decay **(C)** or retained intron **(D)**.

As multiple analyses have pointed to the use of isoforms that do not result in proteins (such as retained introns) or that have small proteins that would likely undergo NMD, we performed an analysis of the small GTPase transcript biotypes. Within GTEx, tissues show a variable usage of both NMD (Figure 7A, top) and retained intron (Figure 7B, top) transcripts, with the brain cerebellum and cerebellar hemisphere with the highest usage within both types. Interestingly, the liver has several samples with the highest usage of NMD transcripts but slightly less usage of retained intron transcripts (Figures 7C,D, top). While tissues like the tibial artery and whole blood are low in both, the putamen has higher NMD usage, and the pituitary higher retained intron (Figures 7C,D, top). Analysis of the blood PAXgene tube datasets shows a higher degree of variability in the use of NMD Figure 7A, bottom) and retained intron (Figure 7B, bottom) transcripts than within GTEx. While analyzing the details of these BioProjects, we realized that methodology explains a large portion of this variability. We used nine different BioProjects (Figures 7C,D, bottom) to highlight this.

BioProject PRJNA746233 is from patients with multisystem inflammatory syndrome in children and was generated with SMARTer Stranded Total RNA-Seq and shows some of both NMD and retained intron transcripts within samples. PRJNA542815 is a small non-coding RNA study for *Mycobacterium tuberculosis* infections that shows high levels of retained intron transcripts and several samples with NMD transcript elevation. PRJNA544474 is a microRNA (miRNA) study for myasthenia gravis patients, PRJNA286878 is a broad miRNA study, and PRJNA380819 is a miRNA study for antidepressant use, where all three show variable levels of both biotypes with several samples very high. In contrast, those studies generated with polyA capture (PRJNA329148-idiopathic pneumonia, PRJNA430406-broad Analysis, PRJNA400331-tuberculosis) show low levels of both biotypes. One unknown RNA prep where methods are not listed, PRJNA702017-uveitis, has levels of both NMD and retained introns similar to PRJNA746233, suggesting it to be from total RNAseq. This suggests that the method of preparing the RNA libraries heavily influences the annotation of NMD and retained intron transcripts.

**FIGURE 8:**
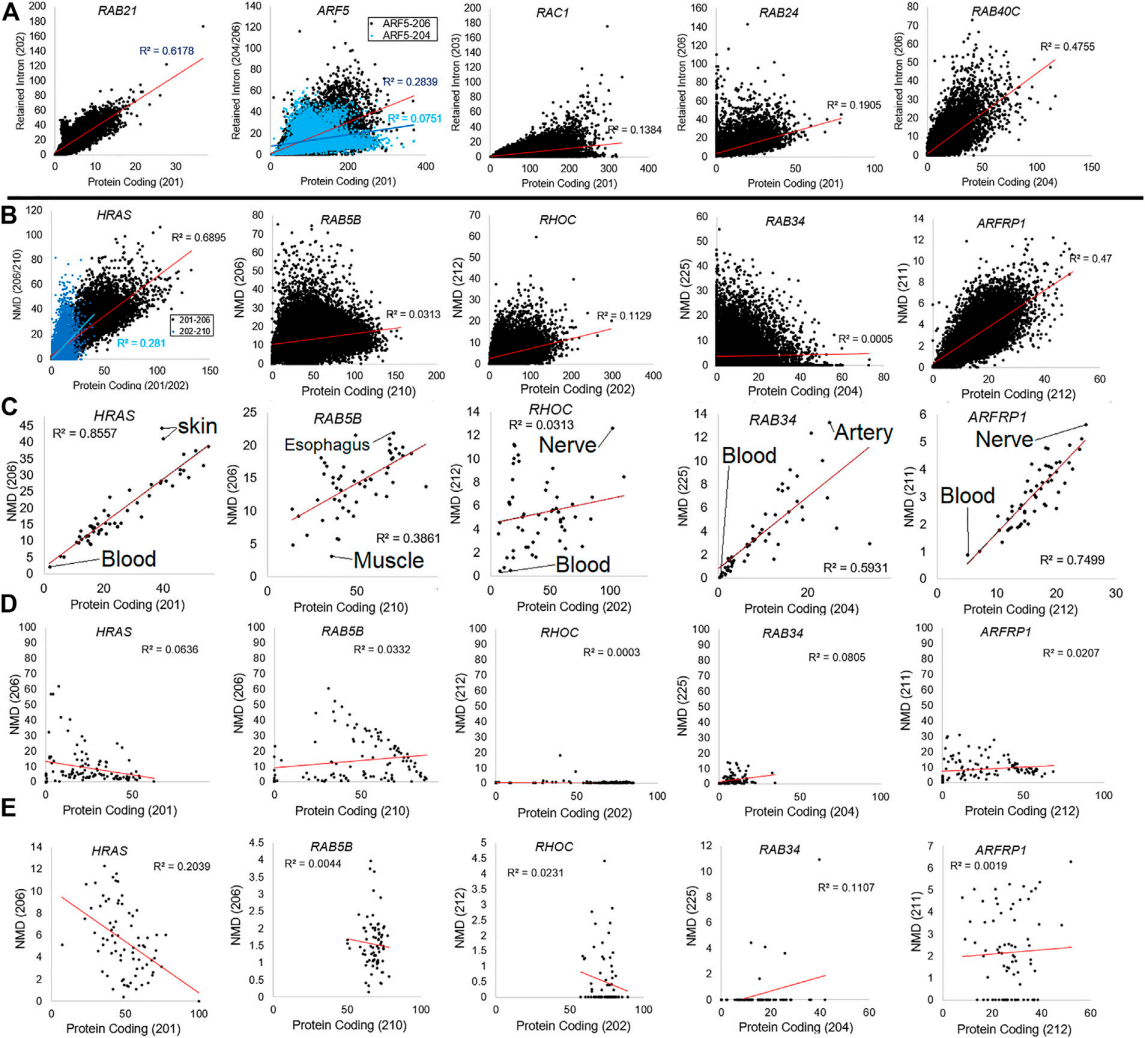
Examples of genes with retain intron or nonsense-mediated decay dynamics. **(A)** Plotting of five representative genes (*RAB21*, *ARF5*, *RAC1*, *RAB24*, and *RAB40C*) for the top protein coding isoform (*x*-axis) relative to the retained intron isoform (*y*-axis) for all samples of GTEx. Values are shown as transcripts per million (TPM). The R-squared correlation for each panel’s best fit line is listed. **(B)** Plotting of five representative genes (*HRAS*, *RAB5B*, *RHOC*, *RAB34*, and *ARFP1*) for the top protein coding isoform (*x*-axis) relative to the nonsense-mediated decay isoform (*y*-axis) for all samples of GTEx. **(C)** Compiled value for all samples within a tissue of GTEx with top tissues labeled. **(D)** Analysis of each gene’s percent NMD transcript (*y*-axis) relative to protein-coding (*x*-axis) averaged for each BioProject of “Blood PAXgene” samples. **(E)** Representative data from BioProject PRJNA691933 (our high-density total RNAseq of hospitalized COVID-19 patients) showing each gene’s percent NMD transcript (*y*-axis) relative to protein-coding (*x*-axis) for each sample.

To further support this discovery, we addressed the correlation of retained intron transcripts (Figure 8A) or NMD transcripts (Figures 8B–E) relative to protein-coding transcripts for five different genes. Analysis of the GTEx data for retained introns of five genes shows that in some cases (*RAB21*- R-squared 0.6178, *RAB40C*- 0.4755), there is a correlation between the use of retained intron relative to the protein-coding version. However, in others, there is little correlation (*ARF5*-0.2839, *RAC1*-0.1384, *RAB24*-0.1905). The same is true for five common NMD transcripts. *HRAS-206* codes for a 154 AA protein that is predicted to be degraded by NMD and has a frameshift altering the c-terminal region. This transcript is correlated (0.6895 R-squared) with the main protein coding isoform (HRAS-201) across GTEx samples (Figure 8B) and in different tissue (Figure 8C), with skin and brain datasets showing the highest levels of NMD-based transcripts. The other four NMD transcripts show far less correlation. The origin of the tissue for NMD transcripts correlates with the NMD transcript for ARFRP1, where brain and nerve tissue have higher NMD transcript levels. One of the most surprising findings within our Biotype analysis for small GTPases was variation within tissues of GTEx. The significant differences between tissues such as blood and the brain are striking. It has been noted that the expression of microRNA such as miR-128, which is brain-specific, can suppress NMD (Bruno et al., 2011) by regulating the essential NMD factor SMG1 (Wang et al., 2013). The balance of NMD in the brain is still critical, as NMD dysregulation can result in neurodevelopmental disorders (Jaffrey and Wilkinson, 2018).

In nearly all of the NMD transcripts, blood has the lowest NMD-based levels. A more detailed analysis of the blood PAXgene tube samples shows no correlation between the protein-coding and the NMD transcript (Figure 8D). We performed further analysis of the highest density sequencing project of blood PAXgene samples (Prokop et al., 2021), which focused on the total RNA signatures in hospitalized COVID-19 patients relative to controls (BioProject PRJNA691933, Figure 8E). This dataset shows little correlation between the protein-coding transcript. Still, it shows some samples with high NMD transcripts, suggesting future work is needed to define why NMD transcripts can be elevated within individuals.

NMD is a process within cells that protects from dominant-negative, partial inhibition, or gain-of-function smaller versions of the protein through the degradation of products where ribosomal proteins do not remove exon-junction accumulating proteins due to early termination (Chang et al., 2007). NMD thus contributes heavily to human health genetics (Holbrook et al., 2004), including cancer (Lindeboom et al., 2016). Through the use of total RNAseq and variant screening in the RNA, our group has previously discovered that NMD inhibition through viral infections can result in the accumulation of NMD-based transcripts that change cellular outcomes (Prokop et al., 2020), where a dominant negative variant can be reactivated by virus to cause a rare transient disease within the cells of viral infection, termed viral-induced genetics (Prokop et al., 2022). Many of the blood samples with the highest levels of NMD transcripts throughout this study had bacterial or viral infections, suggesting that environmental factors may modify NMD transcript levels of small GTPase genes.

The consistent presence of highly expressed NMD transcripts suggests that not all these transcripts are being degraded as initially thought. Even with a premature stop codon (PSC), many of these transcripts contribute to a protein product still highly expressed in the body. In genes with a high number of alternatively spliced transcripts, it has previously been shown that these members have multiple NMD-regulated transcripts (Lewis et al., 2003). However, it has also been shown that NMD of these transcripts can have a bias in actual degradation (Hauer et al., 2016). Non-sense-mediated decay transcripts appear to be highly prevalent in the small GTPase family, with 116 NMD predicted transcripts for the small GTPase genes (Figure 1). Over the GTEx samples, *HRAS-206* is the highest expressed NMD transcript, followed by *RAB5B-206*, *RHOC-212*, *HRAS-210*, and *RAB34-225*. Nearly all of the NMD transcripts of the small GTPase family have been seen expressing >1 TPM within a tissue, suggesting that the family has a potential lack of NMD regulation occurring. This has recently been observed in a family with pancreatic ductal adenocarcinoma identified with a *RABL3* truncating variant (Nissim et al., 2019). The truncated variant does not undergo NMD, resulting in a peptide that promotes KRAS prenylation and cancer outcomes. Broader escape of NMD in genes such as *POMP* impacts autoimmunity (Poli et al., 2018), whereas genes involved in checkpoint inhibitors in anti-tumor immunity are clinically beneficial (Litchfield et al., 2020). While some general mechanisms of NMD escape have been proposed (Dyle et al., 2020), a broader analysis of NMD escape within the small GTPase family is needed in the future.

### Genetic variants impacting small GTPase splicing

**FIGURE 9:**
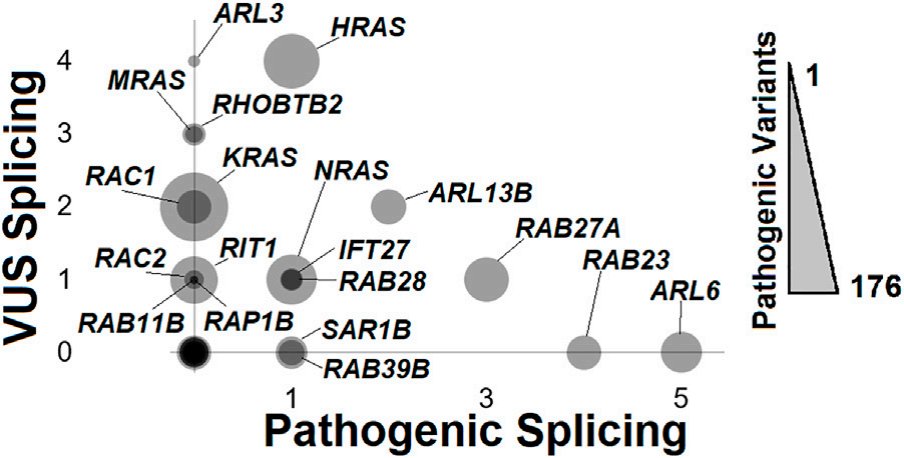
ClinVar mapping of rare variants impacting splicing. The number of pathogenic splicing variants annotated from ClinVar (*x*-axis) relative to the number of Variants of Uncertain Significance (VUS) for splicing (*y*-axis) relative to the overall number of pathogenic variants, including splicing, missense, frameshift, and nonsense (size of bubble) for each of the small GTPase genes. Each bubble with non-zero x- or *y*-axis values is labeled.

Reflecting on NMD transcripts points to the important role of variants within small GTPases to be associated with pathology, notably those impacting splicing. ClinVar is a database of deposited variants for human genes linked to potential human diseases/disorders, where variants are deposited as those potentially pathogenic (including likely pathogenic) and variants of uncertain significance (VUS). Extraction of small GTPase variants (https://doi.org/10.6084/m9.figshare.20371842, Rare Variants LoF-Splice tab; https://doi.org/10.6084/m9.figshare.21381900.v1 tab ClinVar variants) identifies 20 genes with one or more splice variants (Figure 9). *ARL6*, associated with autosomal recessive Bardet-Biedl syndrome 3 (OMIN# 608845), has the most identified pathogen splice variants. This is followed by *RAB23* (Carpenter syndrome, OMIM# 606144), *RAB27A* (Griscelli syndrome, type 2, OMIM# 603868), *ARL13B* (Joubert syndrome 8, OMIM# 608922), *HRAS* (multiple phenotypes, OMIM# 190020), *NRAS* (multiple phenotypes, OMIM# 164790), *IFT27* (Bardet-Biedl syndrome 19, OMIM# 615870), *RAB28* (Cone-rod dystrophy 18, OMIM# 612994), *SAR1B* (Chylomicron retention disease, OMIM# 607690), and *RAB39B* (Waisman syndrome, OMIM# 300774). Genes such as *ARL3*, *MRAS*, *RHOBTB2*, *KRAS*, *RAC1*, *RAC2*, *RIT1*, and *RAP1B* have splicing VUS annotated. This highlights that splicing variants can alter small GTPases resulting in human diseases.

However, common variants can also alter gene splicing and associate with traits. Akin to the way GWAS studies attempt to draw correlations between genetic variants and their associated phenotypes, splicing quantitative trait loci (sQTL) draw correlations between variants that may impact splicing patterns. While studying the GTEx transcripts, it was noted that *GEM* has spliced isoforms that were elevated within individuals across multiple tissues. Yet, these isoforms seem to be highly correlated to the individual, independent of tissue of transcriptomics. Thus, it is an excellent example of explaining a highly penetrant sQTL. *GEM* is a member of the RGK protein Ras subfamily that has a switch II function altering the phosphate-binding site (Splingard et al., 2007) and can inhibit Rho kinase through interactions with ROK alpha and beta (Ward and Kelly, 2006). The expression of *GEM* has been associated with cancer cell lines, with modulation in treatment strategies (Leone et al., 2001).

**FIGURE 10:**
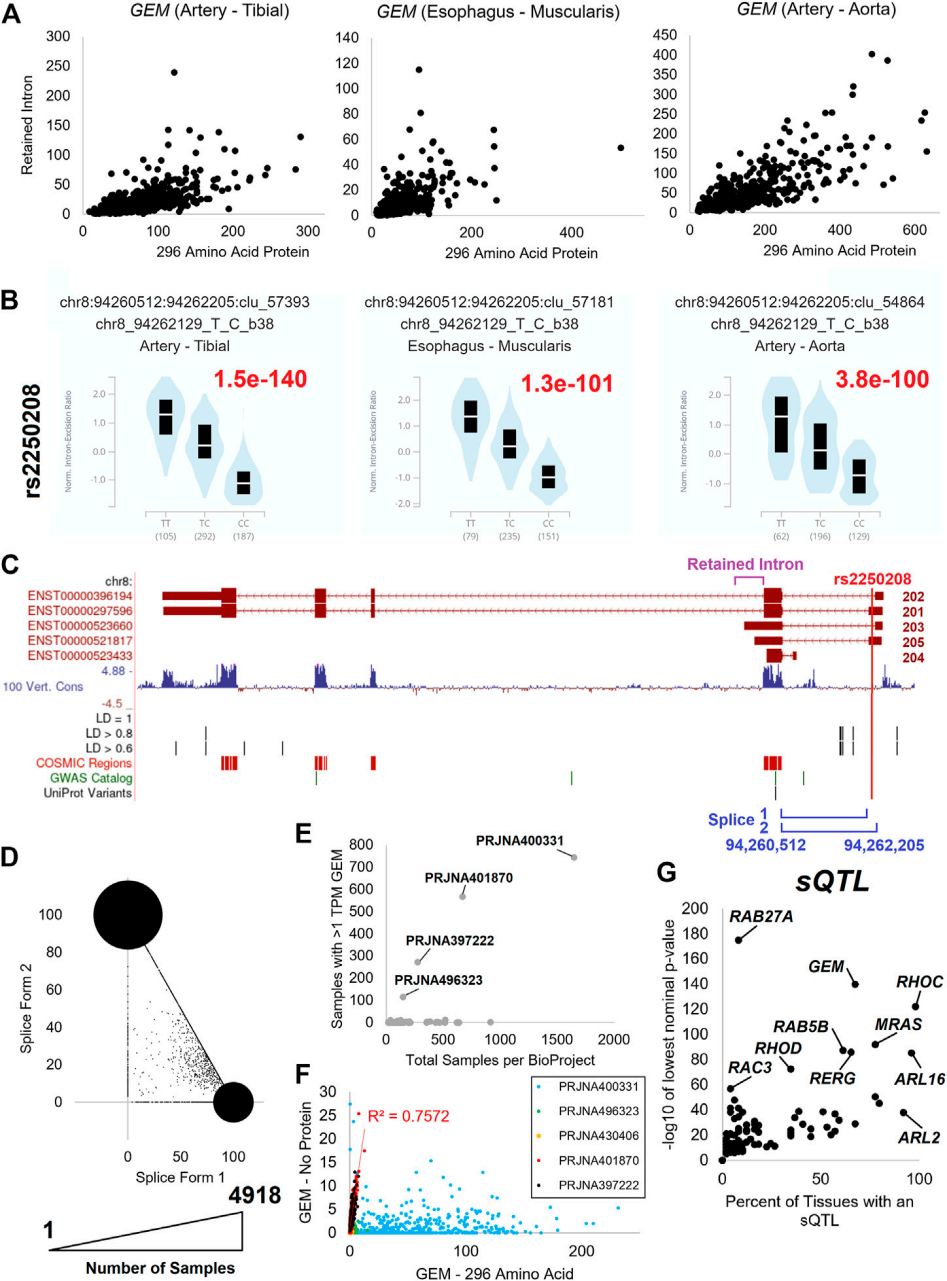
*GEM* isoform dynamics. **(A)** The expression in transcripts per million (TPM) of GEM 296 AA isoform (*x*-axis) relative to retained intron transcripts (*y*-axis) for tibial artery, muscularis esophagus, and aorta artery. **(B)** GTEx annotated splicing quantitative trait loci (sQTLs) for *GEM* based on rs2250208 variant for each of the three tissues in panel **(A)**. TT and CC are homozygous splicing levels, and TC those individuals heterozygous. The GTEx annotated *p*-value is labeled in red. **(C)** Exon map of GEN showing the location of retained intron (magenta), rs2250208 (red), and the splicing sites (blue). Labeled below the exon map are various annotated genomic features, including rs2250208-based linkage disequilibrium (LD) variants, catalog of somatic mutations (COSMIC) variants, Genome-Wide Association Study variants (GWAS), and UniProt variants. **(D)** Expression of the two splice forms in “Blood PAXgene” samples. The *x*-axis is the percent of transcripts using splice form 1, and the *y*-axis is the splice form 2. The size of the spot corresponds to the number of samples with values. **(E)** Compiled analysis of the expression of GEM transcripts in different BioProjects. **(F)** Expression analysis of GEM protein-coding isoforms relative to retained intron transcripts for the BioProjects identified in **(E)**. **(G)** Extraction of GTEx annotated sQTLs for small GTPase genes. The *x*-axis shows the percent of tissues with an eQTL for the given gene, and the *y*-axis shows the lowest nominal *p*-value from GTEx analysis across those tissues.

*GEM* has a retained intron transcript (Figure 10A) and a splicing difference of exon one (Figure 10B). There are two retained intron transcripts (*GEM-203/205*) that are expressed relative to the two protein-coding transcripts (GEM-201/202) that both code for a 296 AA protein (Figure 10A). Both the retained intron and the protein-coding transcripts have a difference in the exon one splice site that is significantly altered by the presence of the rs2250208 variant within multiple tissues of GTEx (Figure 10B). The rs2250208 is found in the exon one location, extending the size of the five prime untranslated regions (5′UTR, Figure 10C). Analysis of the blood PAXgene tube datasets shows that the expression of splice form one relative to two shows a high density of samples with only one of the forms used and a linear number of samples between the two values (Figure 10D). This suggests that the prominent spots in Figure 10D are homozygous individuals, while the samples with values between the two spots are heterogeneous. This suggests that rs2250208 determines the length of the GEM 5′UTR by altering the splicing to exon 2. GEM can be identified in multiple blood samples (Figure 10E), and there is a high correlation between the retained intron and protein-coding GEM isoforms (Figure 10F). While rs2250208 is not associated with any known biological traits (genetics.opentargets.org/variant/8_94262129_T_C), the role of altering the UTR is not yet explored. Because of *GEM’s* function in regulating calcium ion channels, gene therapy with *GEM* has been proposed as an effective, localized alternative to drug-based calcium channel blockers (Murata et al., 2004). Further work is needed for *GEM* to show which 5′UTR sequence is ideal for gene therapy and if the UTR sequence changes the transcript processing.

**FIGURE 11:**
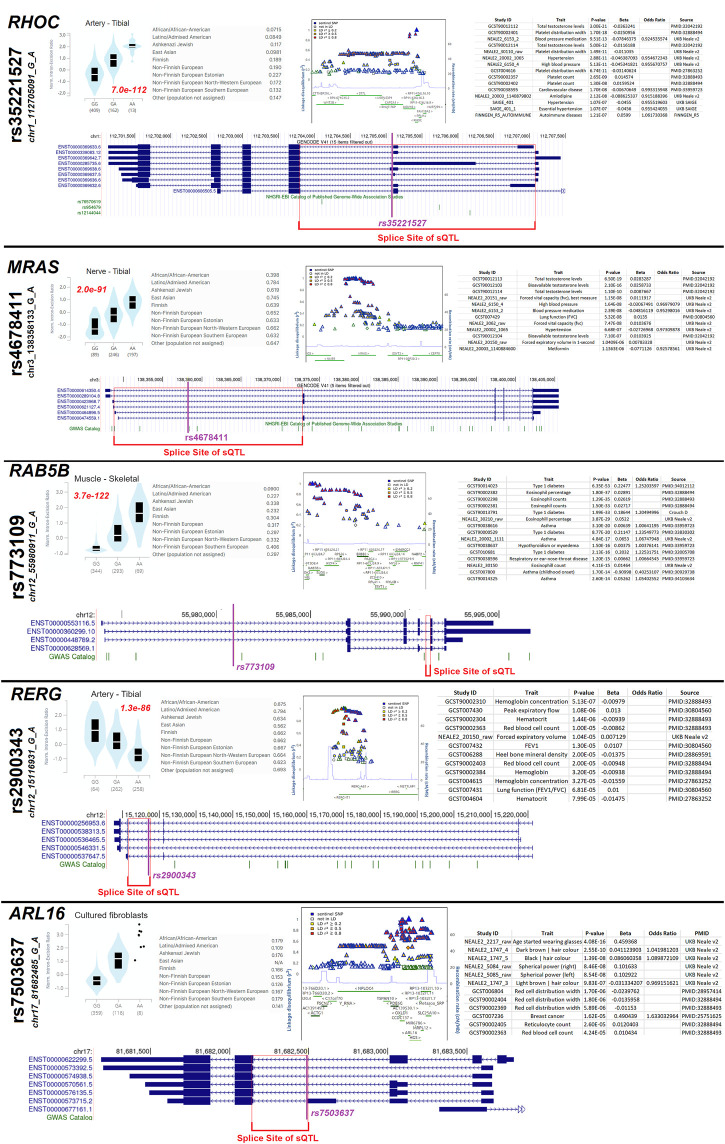
Several highly active sQTL loci. Five (*RHOC*, *MRAS*, *RAB5B*, *RERG*, *ARL16*) splicing quantitative trait loci (sQTLs) containing small GTPase genes are shown as examples. Each gene has the top tissue plot of homozygous or heterozygous splicing levels next to the rsID and chromosome annotated variant, with the red text listing the nominal *p*-value. Next to that plot is the minor allele frequency of the top variant for different populations. Next to allele frequency is the plot of linkage disequilibrium SNPs and a list of Open Targets Genetics annotated traits associated with the variant. Below those panels is an exon map annotating the top variant (magenta) and the splicing site annotated as an sQTL (red).

After identifying the *GEM* sQTL, we further assessed any sQTL within the small GTPase members (Figure 10G, https://doi.org/10.6084/m9.figshare.20371842, sQTLs tab). Several genes (*RHOC*, *MRAS*, *ARL16*, *ARL2*) have an sQTL that is found to influence splicing in multiple tissues, several have sQTLs that impact a few tissues (*GEM*, *RAB5B*, *RERG*, *RHOD*), and a few have significant sQTLs active in a select number of tissues (*RAB27A*, *RAC3*). The significant sQTLs were assessed for phenotypic connections, identifying five sQTLs that may be biologically active (Figure 11).

The variant rs35221527 has an sQTL for *RHOC*, with the tibial artery having the most significant *p*-value (7e-112), which is found with the highest frequency in non-Finnish Europeans with only a few variants in linkage disequilibrium (LD). The variant found in the middle of the splice sites of the sQTL is significantly associated with total testosterone levels, platelet distribution width, and blood pressure/hypertension. The variant rs4678411 is an sQTL for *MRAS* with the most significant *p*-value in the tibial nerve (2e-91), which is found throughout all populations and has many variants in LD. The variant is also found in the middle of the splice site of the sQTL and is associated with testosterone levels and blood pressure. The variant rs773109 is an sQTL for *RAB5B* with the most significance in skeletal muscle (3.7e-122), which is found with the highest allele frequency in non-Finnish southern Europeans with many variants in LD. The lead variant is far from the splice site of the sQTL, suggesting a potential LD variant of function for traits including Type-1 diabetes and eosinophil percentage. The variant rs2900343 is associated with an sQTL in *RERG* that is most significant in the tibial artery (1.3e-86) and found throughout all populations with a complex LD block. The lead SNP falls at the predicted splice site and is associated with hemoglobin concentration. Finally, the variant rs7503637 is associated with an sQTL for *ARL16* that is found in most populations with a complex LD block. The lead SNP is located at the predicted splice site of the sQTL and is associated with wearing glasses and hair color. This suggests that genetic variants influencing small GTPase splicing, whether common or rare, can be associated with altered human biology and potentially disease.

### Sex differences in GTPase splicing and expression

**FIGURE 12:**
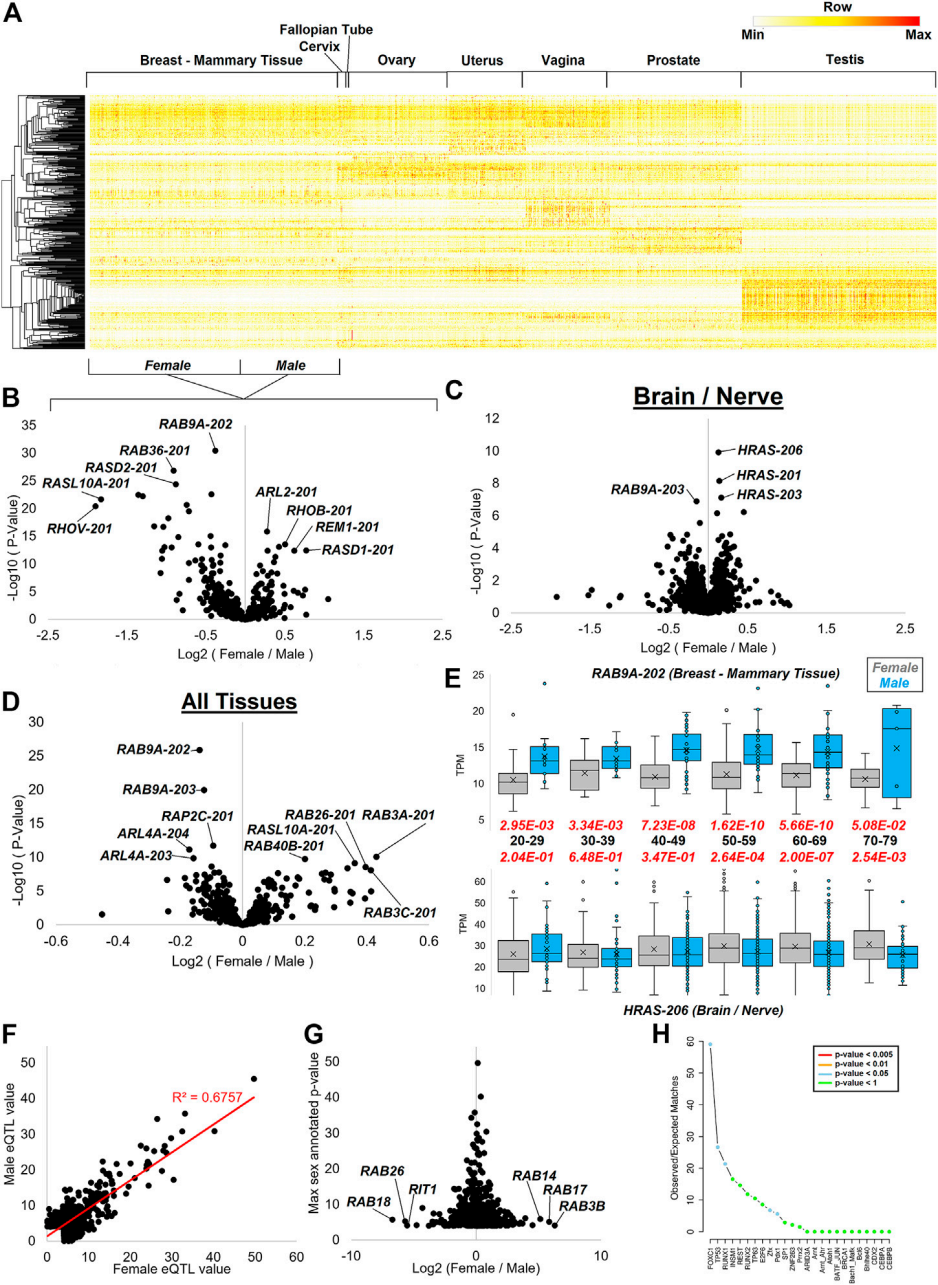
Splicing dynamics within reproductive datasets and sex differences. **(A)** Heat map of all small GTPase isoforms for tissues with sex differences in GTEx. Mammary breast tissue is the only one with both male and female data marked on the bottom for the grouping. Coloring is based on the z-score for each gene, with the middle level in yellow and the highest in red. **(B–D)** Volcano plot with the log2 fold change of female vs. males (*x*-axis) relative to the -log10 *p*-value of female vs. male (*y*-axis) shown for mammary breast tissue **(B)**, multiple brain and neural datasets **(C)**, and all tissues **(D)** of GTEx. **(E)** Representative box and whisker plot of two transcripts showing sex differences at various ages. The top shows the *RAB9A-202* transcript, and the bottom shows *HRAS-206* isoform. The black text in the middle is the age group with the red text the *p*-value of female (gray) vs. male (cyan). **(F)** GTEx annotated sex-based expression quantitative trait loci (sbeQTLs) for small GTPase genes, showing the female −log10 *p*-values on the *x*-axis and male values on the *y*-axis. The red line shows the best fit line and R-squared correlation. **(G)** The log2 fold change of female vs. male *p*-values (*x*-axis) from panel F relative to the top annotated *p*-value. **(H)** SNP2TFBS analysis of the top variant with >2 and <−2 fold change in **(G)**, showing the enrichment of transcription factor binding sites in the SNPs identified.

Several of the sQTLs pointed to a role in sex hormone biology. Therefore, we took a broader mapping of differences between males and females for splicing and expressing small GTPase genes. Tissues of both the male and female reproductive systems have an array of small GTPase isoforms expressed within the GTEx data, giving robust clustering of transcripts for each tissue (Figure 12A).

As the breast tissue is one shared between males and females yet shows a remarkable difference in expression due to hormones such as estrogen and sex chromosomes (Oliva et al., 2020), we selected it to perform Limma (Ritchie et al., 2015) based differential analysis of the small GTPase transcripts (Figure 12B). Multiple transcripts are elevated in female breast tissue relative to males including *ARL2-201* (184AA), *RHOB-201* (196AA), *REM1-201* (298AA), and *RASD1-201* (281AA). Multiple transcripts show male elevation including *RAB9A-202* (201AA), *RAB36-201* (267AA), *RASD2-201* (266AA), *RASL10A-201* (203AA), and *RHOV-201* (236AA). In brain and nerve GTEx samples, *RAB9A* also shows higher levels in males, while multiple transcripts of *HRAS* show higher expression in females (Figure 12C). *HRAS* is broadly expressed throughout brain regions and has been shown to contribute to neuronal differentiation (Park et al., 2016), with downregulation involved in brain gliomas (Lymbouridou et al., 2009). Thus the discovery of the subtle brain differences between males and females may contribute to sex differences in brain development or the 1.6 higher risk of gliomas in males (Carrano et al., 2021).

Analysis of sex differences in all tissues for the small GTPase genes shows an extensive list of transcripts (Figure 12D). These include *RAB3A-201* (220AA) and *RAB3C-201* (227AA), both RAB3 genes. These genes are highly involved in neurotransmitter exocytosis, calcium-ion-triggered release of neurotransmitters, spontaneous secretion, regulation of neurotransmitter transport and secretion, and hormone secretion (Schlüter et al., 2002). RAB3 gene family expression is fairly high within the hypothalamus and pituitary tissue along with sex organs such as the prostate, testis, and ovary. *RAB3A* is a central gene involved in neurotransmitter release (Geppert et al., 1994) through synaptic vesicle fusion (Geppert et al., 1997). *RAB3C* is involved in synaptic vesicle exocytosis (Fischer von Mollard et al., 1994). *RAB3A* involvement in secretory cells exocytosis (Holz et al., 1994) and *RAB3C* in catecholamine secretion (Su et al., 1994) suggest that this sex difference may modulate many hormones and endocrine differences noted between males and females.

The repeated measure of multiple *RAB9A* transcripts has significantly higher expression in males than females and is found on chromosome X, warranting an analysis of age influence on expression between sexes. *RAB9A-202* shows significant sex differences at all ages, with the most significance seen from 50–69 years of age and less separation at 70–79 years of age (Figure 12E). A similar trend is seen for *HRAS-206* in brain and nerve tissues. However, *HRAS* shows higher levels in males at age 20–29, contrary to all other ages. Along with other RAB genes found on chromosome X, *RAB9A* is associated with autophagy control (Shang et al., 2021). The protein’s role in endosomes (Mahanty et al., 2016) and its broad expression suggest a broad potential role in sex differences. However, the knockout of *RAB9A* in mice is not associated with any significant phenotypic changes (Meehan et al., 2017). The gene has not been connected to any human pathologies based on OMIM. Several recent studies have suggested a possible role of *RAB9A* in oncogenic risks (Sun et al., 2020; Zhu et al., 2020), which may warrant future analyses on the role of sex differences for *RAB9A* in cancer.

The robust nature of GTEx to identify sex differences and variants that drive the expression of quantitative trait loci (eQTLs) make it possible to determine sex-based eQTLs (Oliva et al., 2020). Extraction of the sex-based eQTLs (https://doi.org/10.6084/m9.figshare.20371842, sbeQTLs tab) for small GTPases shows most variants associated with changes in expression to the genes to be correlated in males and females (Figure 12F). Differences in these variants between males and females show higher significance in females for *RAB14*, *RAB17*, and *RAB3B* eQTLs, while males have higher significance for *RAB18*, *RAB26*, and *RIT1* (Figure 12G). Further processing of the variants associated with the eQTLs for potential altered transcription factors reveals a significant enrichment for FOXC1, TP53, and RUNX1 binding alterations (Figure 12H), all of which are associated with cancer.

### Splicing changes in cancer patients

**FIGURE 13:**
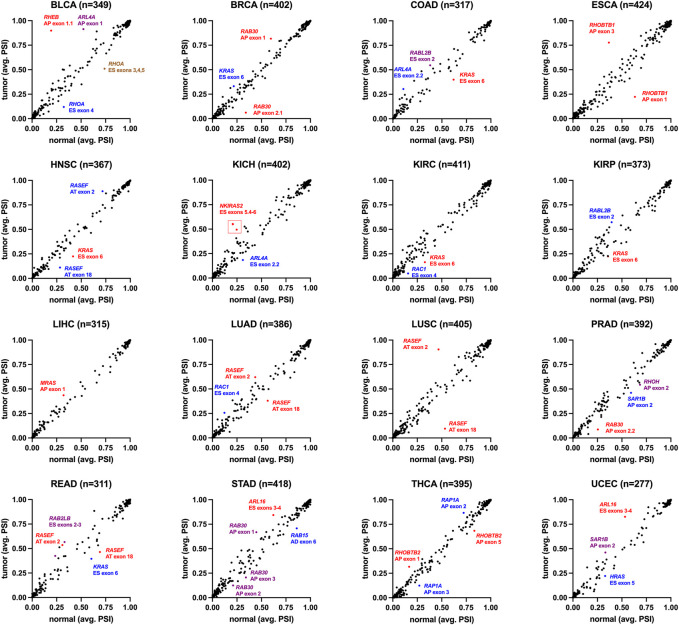
Small GTPase splicing dynamics in human cancer. Percent-spliced-in (PSI) values are the normalized read counts for inclusion of the splicing event over the normalized total (inclusion and exclusion) read counts for that event. The small GTPase splice isoforms were selected, and their PSI values averaged for all normal (*x*-axis) and tumor tissue (*y*-axis) within the indicated cancer types: BLCA-bladder urothelial carcinoma (19 cases/416 controls), BRCA-invasive breast carcinoma (113/1104), COAD-colon adenocarcinoma (41/467), ESCA-esophageal carcinoma (13/190), HNSC- head and neck squamous cell carcinoma (43/511), KICH- kidney chromophobe (25/76), KIRC- kidney renal clear cell carcinoma (72/543), KIRP- kidney renal papillary cell carcinoma (32/300), LIHC- liver hepatocellular carcinoma (50/381), LUAD-lung adenocarcinoma (59/524), LUSC- lung squamous cell carcinoma (49/511), PRAD-prostate adenocarcinoma (52/507), READ-rectum adenocarcinoma (10/176), STAD-stomach adenocarcinoma (37/425), THCA-thyroid carcinoma (71/527), UCEC- uterine corpus endometrial carcinoma (35/555). Listed for each cancer type is the number of isoforms in small GTPases (n). Select splice isoforms are indicated by gene, exon(s), and type of event (AP, alternate promoter; AT, alternate terminator; ES, exon skip).

As small GTPases are highly associated with cancer biology, an analysis of splicing differences in cancer vs. controls is essential. We analyzed the splicing of small GTPase genes through the Cancer Genome Atlas (TCGA) for sixteen cancer forms (Figure 13). In bladder urothelial carcinoma (BLCA), splicing alterations through exon inclusion to *RHEB* and *ARL4A* are elevated in cancer. In contrast, *RHOA* exon inclusion is decreased, representing the most extensive changes in the analysis. *RHEB* expression levels are associated with bladder cancer (Tigli et al., 2013). *RAB30* exon changes are noted in invasive breast carcinoma (BRCA), with a previously established role in triple-negative breast cancer (Dong et al., 2018). *RHOBTB1* has exon changes noted in esophageal carcinoma (ESCA) and thyroid carcinoma (THCA), where it has been associated with cancer cell invasion (McKinnon and Mellor, 2017). *RAP1A* was noted to have an alternative exon in thyroid carcinoma (THCA), where this splicing alteration has been noted in thyroid cancer (Huk et al., 2018).

**FIGURE 14:**
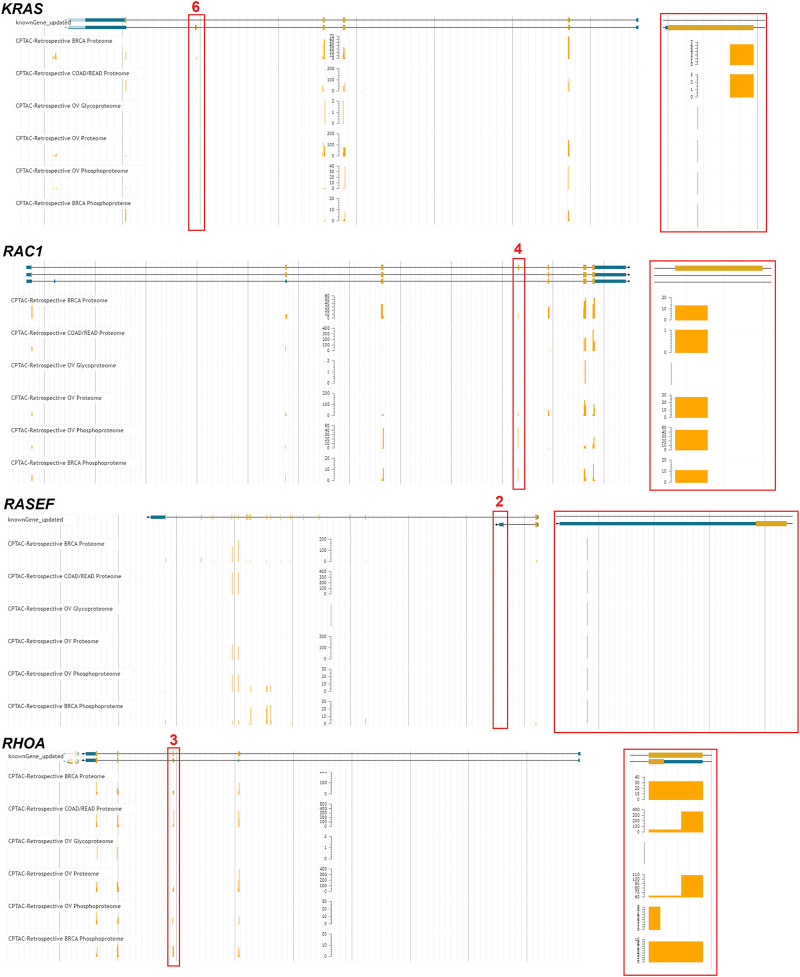
Proteomic analysis of exon skipping in cancer samples. Four genes (*KRAS*, *RAC1*, *RASEF*, *RHOA*) with identified exon skipping were processed through the TCGA peptide genome mapping tool (https://pdc.cancer.gov/jbrowse/). Exons noted in Figure 13 were marked in red, with a zoom in on that exon shown to the right. The yellow bars represent the amount of peptide mapped in six different proteomic datasets.

Several genes were noted in multiple cancer types to have altered exon inclusions. *RASEF* has exon changes noted in head and neck squamous cell carcinoma (HNSC), lung adenocarcinoma (LUAD), lung squamous cell carcinoma (LUSC), and rectum adenocarcinoma (READ). *RASEF* has been identified as a tumor suppressor (Maat et al., 2008), is a potential biomarker for lung cancer (Oshita et al., 2013), is associated with hormone receptor levels in cancer (Shibata et al., 2018), and has been identified to associate with better cancer prognosis (Yu et al., 2019). *RAC1* exon four was noted in multiple cancer types, including kidney renal clear cell carcinoma (KIRC) and lung adenocarcinoma (LUAD). Exon 4 for *RAC1* corresponds to the 192AA vs. 211AA proteins discussed in Figure 6. *KRAS* exon 6 shows up with altered inclusion in colon adenocarcinoma (COAD), head and neck squamous cell carcinoma (HNSC), kidney renal clear cell carcinoma (KIRC), kidney renal papillary cell carcinoma (KIRP), and rectum adenocarcinoma (READ). Exon 6 for *KRAS* corresponds to the 189AA vs. 188AA proteins discussed in Figure 6. The splicing changes of *KRAS*, *RAC1,* and *RHOA* can be observed with variable peptide levels within TCGA proteomic datasets (Figure 14). However, the RASEF alternative stop codon levels cannot be mapped with these proteomic datasets. Including *RAC1* and *KRAS* in our list is a positive sign as their splicing are well-known in cancer, suggesting that some discoveries, such as *RASEF* hold promise but require further analysis.

### RNA modifications and isoform dynamics in hypoxia

**FIGURE 15:**
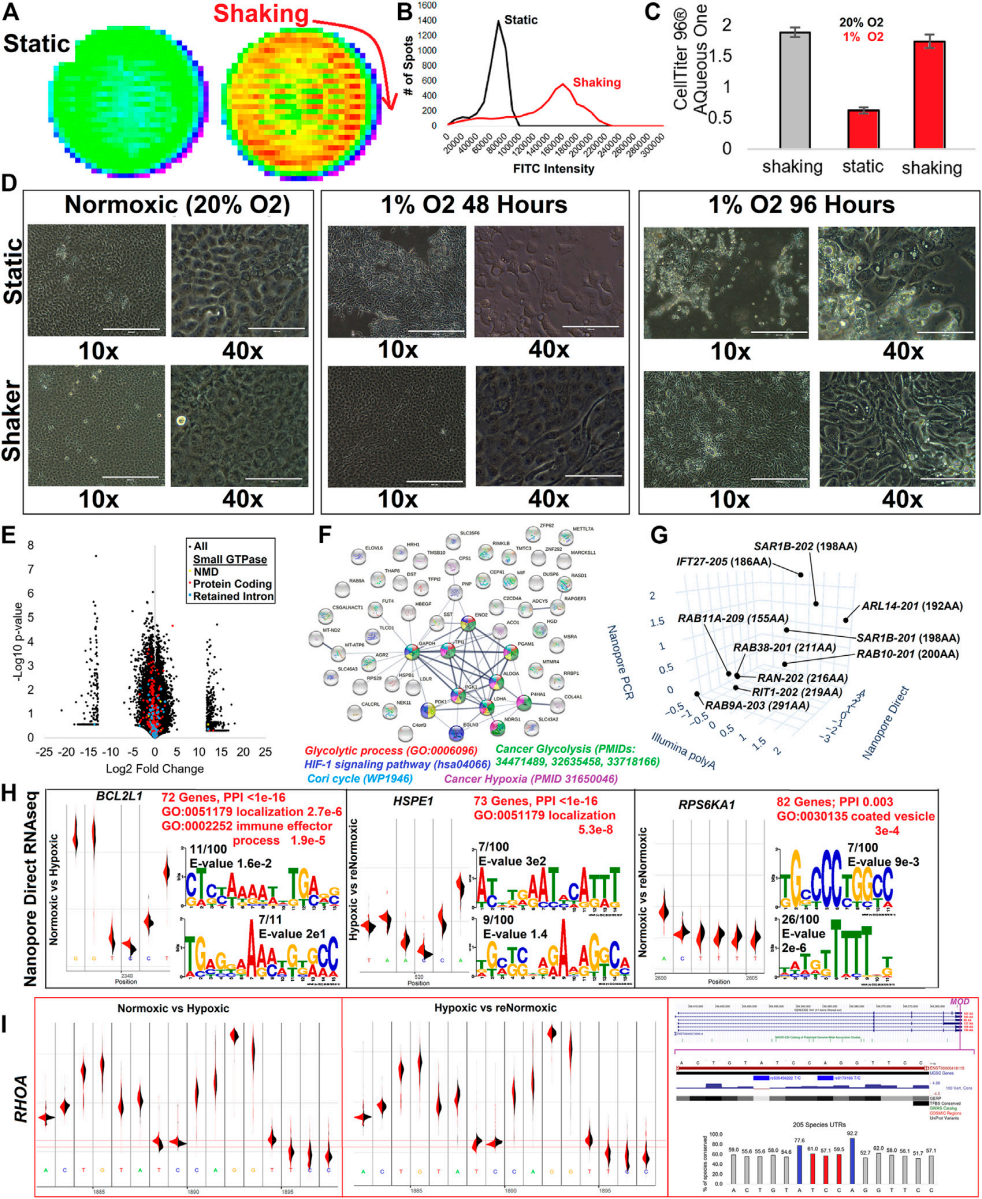
RNA modifications to the multiple isoform UTR of RHOA. **(A)** Representative FITC-Albumin uptake assay in a well of a 6-well plate for cell static or under shaking based on sheer stress for Renal Proximal Tubule Epithelial Cells immortalized with TERT1 (RPTEC-TERT1). **(B)** Analysis of **(A)** with the number of spots measured for various FITC intensities. **(C)** The number of cells attached to the surface as determined by CellTiter 96 AQueous One measurement for cells under normoxia (20% O_2_) and shaking (gray) or in hypoxia (1% O_2_ for 48 h, red) either static or shaking. Error bars represent the standard error of the mean for 96 samples of each condition. **(D)** Light microscopy for cells at 10X or 40X under static or shaker conditions at normoxia (20% O_2_), 1% O_2_ for 48 h, or 1% O_2_ for 96 h. **(E)** Volcano plot of Illumina polyA RNAseq for normoxic vs. hypoxic RPTEC under sheer stress. Only isoforms with an average of 1 transcript per million (TPM) are shown. Small GTPase isoforms are colored for lncRNA (cyan), nonsense-mediated decay (yellow), protein-coding (red), and retained intron (blue). **(F)** STRING plot of genes having an isoform altered in Illumina polyA, Nanopore direct, and Nanopore PCR. Colors correspond to labels shown below for significantly enriched ontology terms. **(G)** Small GTPase isoforms showing altered expression in all three datasets. Each axis shows the log2 fold change of normoxic vs. hypoxic cells from the three datasets. **(H)** Differential analysis of base pair voltage for normoxic cells vs. hypoxic (left), hypoxic to renormoxic (middle), or normoxic to renormoxic (right). For each panel, the top mapped modification location is shown with density maps of the two groups (black vs. red). This is followed by red text indicating the number of genes with significant modifications mapped, enrichment of protein-protein interactions (PPI) based on STRING analysis, and top gene ontology (GO) terms enriched in the gene list. Below that is the top two Meme sequences of modification sites. **(I)** The identification of the *RHOA* modification site changed in normoxic vs. hypoxic conditions and returned to a similar voltage following renormoxia. The far right panel shows the exon structure with the modification site marked magenta. Below that is variant and conservation data for the modification regions. The bottom of the panel shows the conservation of bases based on 205 species sequences, with those in blue the locations of known human variation and those in red showing the altered voltage signal.

Our final analysis involved processing one of the most well-studied environmental alterations of gene transcription, hypoxia. Previously, we developed a sheer-stress culture system using TERT1 immortalized renal-proximal tubule epithelial cells (RPTEC-TERT1) that elevates actin networks to increase cell attachment during hypoxia (Keele et al., 2021). Cells under sheer stress show an increased ability to uptake albumin (Figures 15A,B), a primary role of RPTEC within the kidney tubule. Cell assays, following shaking derived sheer-stress, show retention of cells relative to static conditions (Figure 15C), with morphology significantly altered in the hypoxic cells under sheer-stress (Figure 15D). Illumina polyA captured RNAseq of control and hypoxia-exposed cells under sheer-stress, showed multiple small GTPase transcripts significantly altered (Figure 15E). Two different Nanopore-based RNAseq experiments were performed for long-read transcripts, PCR amplified cDNA, or directly analyzing RNA reads. All isoforms in the same direction as the Illumina polyA significant gene list were identified, finding an enrichment of genes involved in glycolytic process, HIF1 signaling, the Cori cycle, cancer glycolysis, and cancer hypoxia (Figure 15F). Ten of these transcripts were of small GTPases (Figure 15G).

One of the most exciting aspects of direct Nanopore RNAseq is the ability to look at subtle voltage differences between control and hypoxia-exposed cells, suggesting when there are covalent changes to RNA bases transcriptome-wide. Comparisons of normoxic control relative to hypoxic cells identified 72 significant modified transcripts enriched for localization and immune effector processes (Figure 15H). We performed direct Nanopore RNAseq on a third group exposed to hypoxia, then allowed to stabilize for 48 h in normoxic conditions. These renormoxic cells had 73 gene modifications enriched for factors of localization, many of which were those returning to control levels seen altered in hypoxia. This suggests that the covalent changes are transient. Eighty-two genes are altered for normoxic relative to the renormoxic conditions. To the best of our knowledge, this is the first transcriptome-wide analysis of hypoxia-induced transient RNA base editing.

One of the genes identified with a modification in hypoxia that returned to average voltage following re-oxygenation was that of *RHOA* (Figure 15I). The modification site falls on the 3′UTR included in all known *RHOA* transcripts. The nucleic acids near the voltage shift are relatively conserved (>50%) in 206 vertebrate species analyzed, with human variants at two of the flanking residues (rs535456222 and rs8179166). Both variants are rare. The interaction of *RHOA* and hypoxia through the potential modification of the 3′UTR is an exciting observation in that *RHOA* is increased in activation within hypoxia (Chi et al., 2010; Resta et al., 2010; Gilkes et al., 2014). As these cells have an epithelial-to-mesenchymal (EMT)-like transition, it is also interesting to note the incredible overlap that hypoxia-induced metastasis has been suggested to function through the *RHOA* axis (Raheja et al., 2011; Yang et al., 2022). As *RHOA* is a master regulator of tumor cell invasion and metastasis (Chan et al., 2010; Gulhati et al., 2011; Struckhoff et al., 2011), the discovery of the 3′UTR hypoxia-induced RNA base editing to *RHOA* represents an incredible potential future endeavor for small GTPase transcript biology.

## Conclusion

The literature around splicing for small GTPase family members revolves around a few genes, namely *KRAS* and *RAC1*. Yet, we show that splicing dynamics are found throughout the family and that the resulting isoforms are broadly expressed. Both long-read sequencing from Nanopore and short-read sequencing of Illumina have shown the presence of alternatively spliced isoforms. Numerous isoforms change the resulting protein sequence, yet the biological function of these derivative proteins has been poorly characterized for most of the alternative transcripts. Moreover, we have elucidated a remarkable bias of RNA isolation and library prep on capturing transcripts with retained intron and those suspected of undergoing NMD relative to the protein-coding transcripts. As many groups perform RNAseq by summing transcripts to the gene level, we speculate that discovery bias has been expanded for protein-coding versions of the small GTPase family. It is critical to account for this bias and to begin a more extensive focus on individual transcripts for the family instead of gene summation and inference to protein outcomes.

Four of the most exciting findings within this work revolve around genetics and environmental alterations. First, the association of common sQTLs and rare variants near splice sites, which change splicing with high penetrance, to be linked to biological traits suggests that splicing dynamics within the family are likely to manifest biological outcomes such as phenotypic traits and diseases/disorders. Second, several small GTPase members’ role in having differences between males and females, such as *RAB9A* and the *RAB3* members, suggests an under-explored role in how the family can manifest sex differences in cellular processes. Third, while observing the well-studied role of splicing differences for *RAC1* and *KRAS* in cancer patients, we discovered several other recurring differences in genes, including multi-cancer-associated *RAMSEF* splicing change. Finally, using cutting-edge Nanopore sequencing of RNA transcripts without undergoing any cDNA conversions has confirmed ten small GTPase transcripts differentially expressed due to hypoxia while elucidating a 3′UTR RNA modification within the *RHOA* transcripts. This is an exciting discovery with RHOA’s connections to hypoxia and EMT.

As laid out within this report, we suggest several novel areas of future small GTPase research revolving around better exploring splicing and transcript-level biology. For a well-studied family, the amount of knowledge gained through these explorations is surprising, which may hold many promises for oncology, precision medicine, and cellular physiology. In conclusion, it seems likely that the insights of transcript biology for the small GTPase field will continue to resolve many additional insights that can be applied throughout human genetics and biology.

## Material and methods

### Sequence analysis, database extractions, and protein modeling

UniProt details and sequences for all isoforms were extracted using human, reviewed, small GTPase superfamily annotation on 27/06/2022. The Human Protein Atlas (HPA) expression data was extracted on 25/05/2022. The final list of genes used was annotated in UniProt and HPA. All tissue, single cell annotation, and cell line expression for these genes from HPA were extracted for Spearman’s Rank correlation analysis. All heat maps and correlation analyses, including dendrograms, were generated with Broad’s Morphius tools (software.broadinstitute.org/morpheus/). Phylogenetics of the UniProt annotated isoforms (265 sequences from 162 genes) for small GTPases was performed using the Maximum Likelihood method based on the JTT matrix-based model (Jones et al., 1992), using 500 bootstraps for branch clustering. Any sequence alignments were performed with ClustalW (Thompson et al., 1994). Data from NCBI ClinVar (Landrum et al., 2016) was extracted on 17/07/2022 for rare variants. Using the UniProt first annotated transcript, protein modeling of selected small GTPases was performed using a merge of five protein databank (PDB) structure models using YASARA homology modeling (Krieger et al., 2002). Analysis of genomic coordinates was performed using the UCSC genome browser (Navarro Gonzalez et al., 2021) using hg38 annotations. Data from The Cancer Genome Atlas (TCGA) were analyzed by SpliceSeq (Ryan et al., 2016). Cancer types with available normal tissues were downloaded from the MD Anderson Cancer Center bioinformatics database.

### GTEx isoform analysis

Open access data from GTEx (GTEx Consortium, 2020) was extracted on 27/06/2022 through gtexportal. org/home/datasets using version 8 data. These include the normalized transcript per million (TPM) nanopore transcripts (quantification_flair_filter.tpm.txt.gz), sample details (GTEx_Analysis_v8_Annotations_SampleAttributesDS.txt and GTEx_Analysis_v8_Annotations_SubjectPhenotypesDS.txt), transcript TPM for tissues (GTEx_Analysis_2017-06-05_v8_RSEMv1.3.0_transcript_tpm.gct.gz), splicing quantitative trait loci (sQTLs, GTEx_Analysis_v8_sQTL.tar), and sex-biased expression quantitative trait loci (sbeQTLs, GTEx_Analysis_v8_sbeQTLs.tar.gz). The small GTPase genes annotated above from UniProt and HPA were extracted from each dataset. Transcript annotations, including transcript number and biotype, were extracted from the Gencode version 26 annotations (Frankish et al., 2019). Splicing maps and isoform expression images were modified from the GTEx pages for the genes. For sQTL and sbeQTL analyses, linkage disequilibrium was extracted using SNiPA (Arnold et al., 2015), population allele frequencies from gnomAD (Karczewski et al., 2020), genome or phenome-wide association studies (GWAS/PheWAS) from Open Targets Genetics (Ghoussaini et al., 2021), and transcription factor binding sites with SNP2TFBS (Kumar et al., 2017).

### Blood PAXgene tube analysis

The NCBI SRA was quired for “Blood PAXgene” RNA analysis for samples with >5M reads and BioProjects with >20 samples, which included PRJNA679331 (102 samples), PRJNA774204 (20 samples), PRJNA777562 (20 samples), PRJNA787298 (24 samples), PRJNA803436 (202 samples), PRJNA816146 (34 samples), PRJEB10325 (28 samples), PRJNA201039 (69 samples), PRJNA246060 (26 samples), PRJNA267697 (129 samples), PRJNA286878 (43 samples), PRJNA294187 (117 samples), PRJNA294226 (31 samples), PRJNA380819 (516 samples), PRJNA383159 (25 samples), PRJNA385815 (77 samples), PRJNA391912 (191 samples), PRJNA395234 (44 samples), PRJNA418996 (33 samples), PRJNA428989 (45 samples), PRJNA429257 (191 samples), PRJNA434274 (53 samples), PRJNA439269 (120 samples), PRJNA473653 (20 samples), PRJNA492827 (621 samples), PRJNA492829 (639 samples), PRJNA492965 (639 samples), PRJNA494963 (58 samples), PRJNA516650 (22 samples), PRJNA542815 (49 samples), PRJNA544474 (30 samples), PRJNA552470 (197 samples), PRJNA556869 (52 samples), PRJNA565209 (110 samples), PRJNA575507 (24 samples), PRJNA587698 (90 samples), PRJNA596759 (33 samples), PRJNA599020 (45 samples), PRJNA638819 (99 samples), PRJNA648957 (75 samples), PRJNA656180 (128 samples), PRJNA667459 (24 samples), PRJNA669857 (47 samples), PRJNA693831 (27 samples), PRJNA699562 (98 samples), PRJNA702017 (108 samples), PRJNA725183 (32 samples), PRJNA728117 (49 samples), PRJNA768419 (385 samples), PRJNA800337 (119 samples), PRJNA806975 (142 samples), PRJNA647880 (105 samples), PRJNA679264 (201 samples), PRJNA680771 (25 samples), PRJNA683803 (211 samples), PRJNA686397 (195 samples), PRJNA693202 (26 samples), PRJNA702558 (95 samples), PRJNA703029 (70 samples), PRJNA496323 (147 samples), PRJNA705602 (40 samples), PRJNA722046 (69 samples), PRJNA746233 (25 samples), PRJNA756565 (72 samples), PRJNA794277 (128 samples), PRJNA807370 (49 samples), PRJNA343804 (119 samples), PRJNA400331 (1648 samples), PRJNA717662 (152 samples), PRJNA691933 (74 samples), PRJNA693881 (47 samples), PRJNA727526 (95 samples), PRJNA734949 (39 samples), PRJNA735653 (445 samples), PRJNA735656 (186 samples), PRJNA753877 (46 samples), PRJNA762935 (116 samples), PRJNA771014 (69 samples), PRJEB41073 (41 samples), PRJNA201433 (25 samples), PRJNA232593 (45 samples), PRJNA315611 (355 samples), PRJNA327986 (36 samples), PRJNA329148 (26 samples), PRJNA341405 (44 samples), PRJNA352062 (914 samples), PRJNA354367 (24 samples), PRJNA369684 (434 samples), PRJNA378794 (38 samples), PRJNA380820 (40 samples), PRJNA384259 (50 samples), PRJNA390289 (172 samples), PRJNA397222 (275 samples), PRJNA398240 (22 samples), PRJNA401870 (670 samples), PRJNA430406 (37 samples), PRJNA437114 (43 samples), PRJNA454445 (518 samples), PRJNA454694 (64 samples), PRJNA476781 (468 samples), PRJNA493832 (48 samples), PRJNA494155 (51 samples), PRJNA504827 (31 samples), PRJNA511891 (25 samples), PRJNA526259 (98 samples), PRJNA526839 (52 samples), PRJNA533086 (357 samples), PRJNA562305 (49 samples), PRJNA588242 (100 samples), PRJNA600846 (101 samples), PRJNA601661 (38 samples), PRJNA607120 (117 samples), PRJNA630674 (79 samples), PRJNA634938 (76 samples), PRJNA638653 (48 samples), PRJNA639278 (36 samples). All SRA files were downloaded using the SRAtoolkit and processed with Gencode 39 transcriptome (Frankish et al., 2019) using Salmon (Patro et al., 2017) alignment to generate transcripts per million (TPM).

### Renal proximal tubule epithelial cells-TERT1 and nanopore sequencing

Renal proximal tubule epithelial cells immortalized with TERT1 (RPTEC-TERT1, ATCC, #CRL-4031) were grown under sheer stress and hypoxic conditions as previously described (Keele et al., 2021). Previously generated Illumina polyA RNAseq data was also described within that work (Keele et al., 2021). Albumin uptake assays were performed by placing RPTEC-TERT1 into 6-well plates, growing cells until confluent in DMEM:F12 supplemented with RPTEC growth kit (ATCC, #ACS-4007), followed by either sheer stress (150 RPM using a MaxQ CO2 plus shaker) or static conditions for 1 week. FITC-Albumin (Sigma, #A9771) was placed onto cells at 5 mg/ml in RPTEC growth media and incubated for 24 h at 5% CO_2_ at 37°C. Cells were then washed with PBS, and a 30 × 30 matrix was measured in each well for FITC level using a CLARIOstar plate reader (BMG Labtech). Cell proliferation was measured by growing RPTEC-TERT1 cells to confluency in a 96-well tissue culture treated plate followed by shaking (300 RPM using a MaxQ CO_2_ plus shaker) or static conditions for 1 week. Hypoxia was induced at 1% O_2_ using nitrogen within a HCbi incubator for 48 h. Wells were washed with PBS, RPTEC growth media added, and CellTiter 96 Aqueous one solution (Promega #G3581) used according to manufacture recommendations. Light imaging of cells was performed on an EvosXL microscope.

For the generation of Nanopore data, RPTEC-TERT1 cells were grown to confluency in 100 mm plates, held under sheer stress (150 RPM) or static conditions for 1 week, followed by RNA isolation using RNeasy (Qiagen #74104) with QIAshredder. Direct RNAseq was performed using the Nanopore Direct RNA Sequencing Kit (#SQK-RNA002), and PCR amplified with PCR-cDNA Sequencing Kit (#SQK-PCS109), followed by sequencing on Nanopore flow cell (R9.4.1). Analysis of Nanopore mapping data was performed as previously published (Bilinovich et al., 2020). All RNA data is available as fastq (Illumina polyA and Nanopore PCR amplified) or fast5 (Nanopore direct RNAseq) deposited under BioProject PRJNA604721 within NCBI SRA.

## Data Availability

The data presented in the study are deposited in the FigShare repository, accession numbers https://doi.org/10.6084/m9.figshare.20371842, https://doi.org/10.6084/m9.figshare.21381900.v1, https://doi.org/10.6084/m9.figshare.20600697.v1 and within NCBI SRA under BioProject PRJNA604721.
